# Paleoeconomy more than demography determined prehistoric human impact in Arctic Norway

**DOI:** 10.1093/pnasnexus/pgac209

**Published:** 2022-10-07

**Authors:** Tony Brown, Dilli P Rijal, Peter D Heintzman, Charlotte L Clarke, Hans Peter Blankholm, Helge I Høeg, Youri Lammers, Kari Anne Bråthen, Mary Edwards, Inger G Alsos

**Affiliations:** The Arctic University Museum of Norway, UiT, The Arctic University of Norway, N-9037 Tromsø, Norway; The Arctic University Museum of Norway, UiT, The Arctic University of Norway, N-9037 Tromsø, Norway; The Arctic University Museum of Norway, UiT, The Arctic University of Norway, N-9037 Tromsø, Norway; The Palaeolab., Geography and Environmental Science, University of Southampton, Southampton SO17 1BJ, UK; The Institute of Archaeology, UiT, The Arctic University of Norway, N-9037 Tromsø, Norway; Department of Geosciences, University of Oslo, 0371 Oslo, Norway; The Arctic University Museum of Norway, UiT, The Arctic University of Norway, N-9037 Tromsø, Norway; The Department of Arctic and Marine Biology, UiT, The Arctic University of Norway, N-9037 Tromsø, Norway; The Palaeolab., Geography and Environmental Science, University of Southampton, Southampton SO17 1BJ, UK; The Arctic University Museum of Norway, UiT, The Arctic University of Norway, N-9037 Tromsø, Norway

**Keywords:** Arctic ecology, hunter–gatherers–fishers, sedimentary ancient DNA, sustainability, climate human impact

## Abstract

Population size has increasingly been taken as the driver of past human environmental impact worldwide, and particularly in the Arctic. However, sedimentary ancient DNA (sedaDNA), pollen and archaeological data show that over the last 12,000 years, paleoeconomy and culture determined human impacts on the terrestrial ecology of Arctic Norway. The large Mortensnes site complex (Ceavccageađgi, 70°N) has yielded the most comprehensive multiproxy record in the Arctic to date. The site saw occupation from the Pioneer period (c. 10,000 cal. years BP) with more intensive use from c. 4,200 to 2,000 cal. years BP and after 1,600 cal. years BP. Here, we combine on-site environmental archaeology with a near-site lake record of plant and animal sedaDNA. The rich animal sedaDNA data (42 taxa) and on-site faunal analyses reveal switches in human dietary composition from early-Holocene fish + marine mammals, to mixed marine + reindeer, then finally to marine + reindeer + domesticates (sheep, cattle, pigs), with highest reindeer concentrations in the last millennium. Archaeological evidence suggests these changes are not directly driven by climate or variation in population densities at the site or in the region, but rather are the result of changing socio-economic activities and culture, probably reflecting settlers’ origins. This large settlement only had discernable effects on its hinterland in the last 3,600 years (grazing) and more markedly in the last 1,000 years through reindeer keeping/herding and, possibly domestic stock. Near-site sedaDNA can be linked to and validate the faunal record from archaeological excavations, demonstrating that environmental impacts can be assessed at a landscape scale.

Significance Statement Human impacts in the Arctic (70°N) are assessed over 12,000 years from one of the largest site complexes known using plant and animal sedaDNA, pollen, nonpollen palynomorphs (largely fungal spores), and archaeological data. From these data, it is shown that human activities rather than population levels are most important for driving vegetation changes. These activities are connected to the changing marine environment in the Holocene and a switch from marine to terrestrial resources, which, in turn, impacted the terrestrial environment. We show that combining plant and animal sedaDNA, pollen, and archaeology can provide detailed ecological reconstructions and describe shifting socio-ecological baselines, which in this case were determined by culture (including grazing/herding) associated with waves of immigration.

The Arctic is warming up to three times more rapidly than any other biome, and it is critical to understand how human populations have affected and will continue to impact Arctic ecosystem biodiversity (1–3). Estimated human population sizes have increasingly been used to predict past anthropogenic pressure on natural resources, but this is debated (4, 5). In this study, we set out to test this assumption (population = impact) at both the site and hinterland scale. From the land cover side, human population size has previously been used to estimate the extent of land clearance, but there are serious arguments that this is also simplistic because the type and intensity of farming can change culture and environment even without population change (6). Here, we examine this question using an exceptionally rich sedimentary ancient DNA (sedaDNA) lake record, with pollen, nonpollen palynomorphs (NPPs), and charcoal, from close to probably the largest archaeological site complex in the Arctic (Mortensnes, Varanger Peninusula 70.1°N, Fig. 1a). It reveals species-level ecological (plant and animal) change throughout the Holocene. We also test the summed probability distribution (SPD)-based demographic-impact model that has become the favored approach to these research questions (7, 8).

**Fig. 1.**
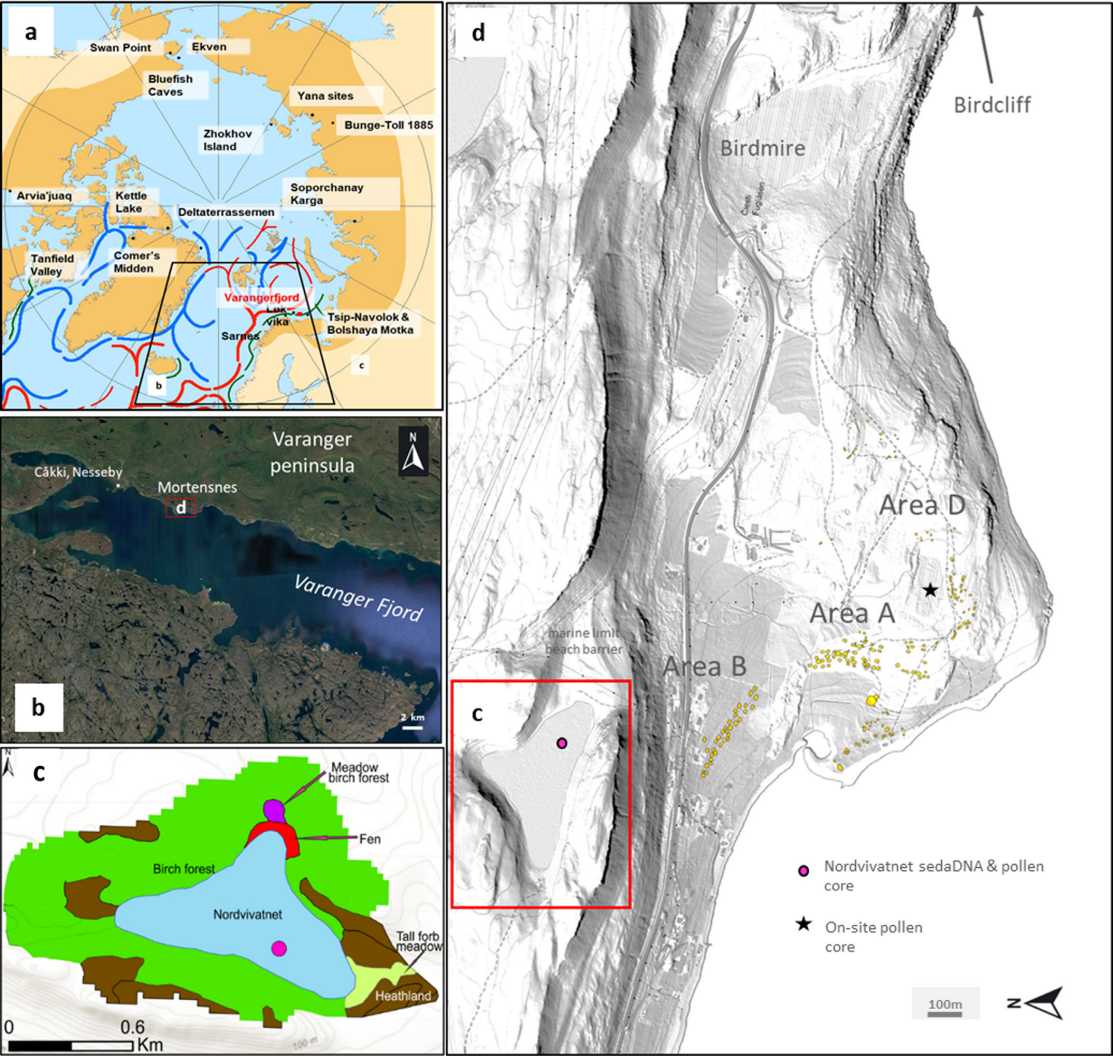
Maps of the area and site. (a) The Arctic circle with principal ocean currents, areas of Arctic vegetation (dark yellow) from (34), and the best known early human colonization sites (various sources), (b) Varanger Fjord with the Mortnesness site area indicated (red rectangle), (c) the vegetation survey around Nordvivannet using types from the Norwegian Vegetation Classification system, and (d) the Mortensnes site complex plan with areas and house areas indicated (yellow dots). Data from Havsforskningsinstituttet (b), Google Earth https://earth.google.com/web/@70.129263,29.00124749,0.07289727a,7700.32164842d,35y,-0h,0t,0r, (c), site data from Kulturminnesøk on Lidar data from Høyedata (d), and from ref. (33) and a survey by Rijal in 2020 (33).

Human colonization of northern Fennoscandia followed the retreating ice sheet (9, 10), with the earliest directly dated archaeological sites being Løkvika and Cåkki (11), both on the Varanger peninsula (Fig. 1). Løkvika is dated by five radiocarbon dates to between 9,920 and 9,640 uncal. years BP and Cåkki is dated to 9,780 uncal. years BP. However, the earliest site on the Varanger peninsula is probably Klubbvik 1 which, on the basis of its altitude and typology (Ahrensburgian affinities), could be in the earliest part of the Holocene, c. 11,700 cal. years BP (11). This site is only 3 km to the east of the site focused on here, Mortensnes (Ceavccageađgi) on the south coast of the Varanger peninsula, and ∼420 km north of the Arctic Circle (Fig. 1). Modern humans entered other areas of the Arctic, including Siberia, this early, and much earlier where there was no ice sheet (12). In North America, a similar pattern is seen with the human crossing of Arctic Beringia probably predating 15,000 cal. years BP (13) whereas the earliest dates for the eastern Arctic seaboard are 13,000 to 10,500 cal. years BP, just after the retreat of the Laurentide ice sheet. As determined from pollen analysis at several northern Norwegian sites, it appears that pioneers were moving along the coast from both west and east (10) where terrestrial vegetation was just re-establishing after the Younger Dryas (14) probably due to excellent hunting and fishing opportunities in open environments. On the Varanger peninsula, prior to the establishment of pioneer birch forest 10,800 to 10,400 cal. years BP the vegetation was shrub-tundra (15). This suggests that hunter–gatherer–fisher colonization of northern Fennoscandia predated, or was part of, the movement north of forest animals particularly reindeer (*Rangifer tarandus*) and moose (*Alces alces*), as well as being related to marine resources. The latter reflect water temperatures and by the earliest Holocene, the Polar Front had returned to close to Svalbard, with the 5° isotherm being close to Varanger (16). Varanger Fjord is a large fjord running almost west–east (50 km) and open to the central part of the Barents Sea, but it is also protected and narrow enough (5 to 10 km) to allow visibility of the land under most conditions. Just outside the fjord runs the southern Barents Sea arm of the North Atlantic Drift/Norwegian Current (Fig. 1a), and to the north Arctic water ingresses from the Arctic Ocean (17). This confluence shelf-area is highly productive, being the junction between Atlantic boreal fish species such as Atlantic cod (*Gadus morhua*) and haddock (*Melanogrammus aeglefinus*), with Arctic cold-water fish such as Polar cod (*Boreogadus saida*) (18).

All the early postglacial colonization sites occur along the coast and suggest colonization from west and east (10). The degree to which the inland areas of the mainland were used, remains unclear as although the terrain was deglaciated, few inland sites date to this period (19). The general view from both traditional artefact-based archaeology and the summed probability distribution of radiocarbon dates (SPD) is that population levels remained low throughout the Older Stone Age (11,500 to 7,000 cal. years BP), started to rise in the Younger Stone Age (7,000 to 3,800 cal. years BP), and rose particularly around 6,000 cal. years BP (8). Population then fluctuated throughout the rest of this period and into the succeeding Early Metal Age (3,800 to 2,000 cal. years BP) (20). Assessing population changes in the Iron Age and Medieval Periods, which are too recent for SPD analysis, is problematic in this area due to the mobility of both the indigenous coastal and eastern Skolt Samí, and an increasing Norwegian population in the last 350 years. By 1801 CE, the population density of the Unjárga–Nesseby municipality (including Mortensnes) was approximately 1 person km^−2^, which is one of the lowest in Europe, although the coastal density population at over 10 persons km^−2^ is comparable with the Troms–Finnmark county as a whole (15 persons km^−2^); thus, it is not atypically low and likely a reflection of waves of inward migration (Statistics Norway, Eurostat). The remarkable concentration of archaeological sites in Varanger (over 100) has attracted research for over a 100 years. The fjord represents a “Maritime Core Area,” where social complexity arose in hunter–gatherer–fisher communities (21–24).

Underlying this and more recent research in western Finnmark (25), is a renewed interest in the relationship of demography to changes in the environment, particularly the terrestrial environment. This has been facilitated by both the production of well-dated high-quality paleoecological datasets along with the accumulation of enough radiocarbon dates to make SPD a statistically valid approach (8, 25). This has been used to argue for both long-term changes in subsistence strategies (9) and a catastrophic population downturn terminating the Gressbakken phase at c. 3,500 cal. years BP that was driven by volcanically induced climate change (25). The effect of volcanic activity has, however, been contested (26). This research postulates a precarious balance of arctic populations and climate but with little reciprocal modification of the environment by these populations. Likewise, ecologists have continued to regard Arctic Fennoscandia as the last great wilderness of Europe, which they consider fundamentally natural, although the region is influenced by reindeer herding through grazing and trampling (27, 28), and potentially highly vulnerable to ongoing human-induced global climate change (29).

Traditionally, ecological and abiotic interactions between climate-vegetation and humans have been studied on short timescales using monitoring and instrumental data, or over millennia using pollen analysis. However, pollen analysis has several limitations in this environment, the two most serious being the pollen source area, which extends far beyond a study site (especially in areas beyond or at the tree line), and the lack of taxonomic precision (30). Although research has addressed the source area problem (31), both limitations reduce the ability of pollen analysis to address site-based questions. A combined approach of sedaDNA alongside pollen can be used to address the inherent limitations of pollen analysis in this environment (32, 33). In this study, we use this approach along with animal sedaDNA, pollen, NPPs (mostly fungal spores), and also a re-analysis of the archaeozoological record from the largest site complex in the European Arctic and its hinterland. This allows us to tackle three questions: (a) How are Holocene subsistence strategies and demography related to terrestrial and/or marine resources? (b) Can we detect any human impact on the hinterland terrestrial vegetation? (c) Is estimated population size a good proxy for this impact in what is the transitional zone between the Boreal and Arctic shrub-tundra biomes? The sedaDNA and one of the pollen diagrams is from a small lake (Nordvivatnet, 4.6 ha), which is the nearest freshwater source to Mortensnes being 200 m north and 75 m above the settlement. We combine this with a new archaeological synthesis of the excavation data also with pollen analysis from within the archaeological site itself. The site also lies in a sensitive ecological zone today at the northern edge of birch-dominated Boreal forest close to the ecotone with the shrub-tundra that covers the northern part of the Veranger peninsula (34).

## Results

### Archaeologic analysis

The archaeological site complex at Mortensnes has been investigated for over a century (35, 36) and is probably the largest site complex in the European Artic. The site is located on both sides of a small promontory (Fig. 1) and covers over at least 1 km^2^ of raised beach, bedrock, scree, and coastal sand-flats. The archaeology covers almost all the archaeological periods and technological traditions of northern Norway/Fennoscandinavia and comprises tent-rings, semi-subterranean pit houses (house depressions, platforms), middens, hearths, graves, a standing stone, and a labyrinth (Supplementary Material Appendix Table S1 and Fig. S1). The site also has an exceptionally large burial ground with several hundred graves in the form of chambers built into scree. The oldest grave dates to c. 3,000 cal. years BP and the cemetery was used for at least 2,500 years (37). From traveler accounts and a lithograph, we know that the site was still inhabited in the late 19th century CE (37). The site complex sits on a staircase of raised beaches from 8 to 70 m asl (41). The settlement areas have traditionally been divided into four zones (A to D) and a cemetery (36). Site A (Karlebotn area) comprises at least 66 houses and 21 probable graves, whilst area B contains 62 houses with an area a short distance to the west (Godluktbukt) with a further 28 houses. Site C has 14 houses, and site D is a smaller, more recent set of houses. The oldest archaeological evidence of human occupation is from Phase I of the older Stone Age (10,000 to 9,000 cal. years BP) and consists of 16 tent rings. These were interpreted as a possible multifamilial family aggregation but could just represent repeated seasonal visitations by small family groups (39). The oldest charcoal dated from the site is 9,305 to 9,883 cal. years BP from site R10, which has eight house-pits (Supplementary Material Appendix Table S1), and this and other dates suggest presence in the later Early Stone Age (early Mesolithic) (40).

The Mortensnes lithic artifact inventory generally follows the standard typological pattern for the Stone Age and Early Metal Age of the region (41), but chronological and/or diagnostic tool-types are relatively few in numbers. The Older Stone Age (c. 10,000 to 5,000 BC) occupations generally contain lots of debitage, some cores, a few retouched blades or flakes, and occasionally tanged points and burins, but, surprisingly few or no scrapers. Oblique, transverse arrowheads occur towards the end of the period. The Younger Stone Age (c. 5,000 to 1,800/1,600 BC) dwellings contain, as a whole and apart from debitage, bifacially retouched (surface-trimmed) points and, made from slate, long, slender Nyelv-points, and single and double-edged knives. The Early Metal Age (c. 1,800/1,600 to 0 BC) dwellings reveal, as a whole and aside from debitage, Sunderøy and Sandbukt bifacially retouched (surface-trimmed) points, and asbestos ceramics (35).

As to technology, the vast majority of the individual older Stone Age occupations belong to the Fosna-tradition (42), which covered the entire Norwegian coast from the beginning of the pioneering period after the last ice age, and which can be traced back to the northwest European Late Glacial Ahrensburg culture. However, one of the occupations at Mortensnes (from R10, and a handful of other sites in the region) from the middle of the Older Stone Age show so-called “Post-Swiderian” affinity rooted in north-eastern Europe (24). This shows that people of the Russo–Finnish or “Eastern” techno-tradition ventured into the Varanger area, although not necessarily carrying a different economic system. As to the Younger Stone Age, the technology rests solidly within the northern Scandinavian slate-oriented technocomplex.

The earliest middens in northern Norway are from Mortensnes, and excavation and faunal analysis of house R12 (Area D, 27 m asl) dated to c. 7,600 to 5,500 cal. years BP (with possible re-use c. 4,300 cal. years BP) revealed evidence of diet, local resources, and hot-stone technology (35, 43). The mammals recovered are dominated by seal (Phocidae) and small rodents, along with beaver, wolf (*Canis*), and whale (Cetacea) but no reindeer (Supplementary Material Appendix Fig. S2). A problem here is that the whale find was one bone; when converted to weight it dominates the assemblage, and it is possible, if unlikely, that it was a stranding. The lack of reindeer has been ascribed to hunting inland (43), but could also reflect marine-resource dominated immigrants from the west (11). The fish are dominated by Atlantic cod (*G. morhua*) and some pollock (*Pollachius virens*) (35, 43). The birds are dominated by black-legged kittiwake (*Rissa tridactyla*), guillemots (*Uria*), and great auk (*Pinguinus impennis*), whilst the more recently analyzed shellfish are dominated by black clam (*Artica islandica*) and periwinkle (*Littorina littorea*) (43). In a quantitative analysis of one 50-cm^2^ quadrat, 77% of the identifiable remains were fish of which 80% were cod (43). This suggests a fishing-based economy using boats, and the exploitation of bird cliffs and the foreshore. There is very little evidence for use of inland taxa, except beaver (also found in the sedaDNA). While wood was almost certainly used in house construction and for fuel, it could have been supplied by driftwood, which is common along this coast today. Excavation of a house in area A (R3), which dates from 4,400 to 2,754 cal. years BP (Supplementary Material Appendix Fig. S2), revealed a rather different faunal assemblage. Here, seal, whale, and reindeer were dominant, with a small number of fish bones of cod and common ling (*Molva molva*) (35, 44). Few birds were recovered and shellfish were not analyzed. The assemblage implies boat fishing, but reindeer utilization suggests more use of the site hinterland. An emphasis on sea mammals is also evident at the Bergeby site, 5 km further to the west and dated 3,925 cal. years BP (44). The third structure (R17, is at 8 m asl and dates from 1,176 to 797 cal. years BP (Supplementary Material Appendix Fig. S2). It reveals a totally different resource spectrum, with the mammals dominated by sheep/goats (*Ovis*/*Capra*), reindeer, cattle (*Bos taurus*), and pig (*Sus scrofa*). There are some seal but no whale. The fish are dominated by very large quantities of cod and haddock (*Melanogrammus aeglefinus*) and the birds are dominated by rock ptarmigan (*Lagopus mutus*), kittiwake, and guillemots. This assemblage implies both summer grazing and the collection of hay and fodder for the overwintering of the domesticated stock. This pastoral farming is combined with reindeer, boat fishing, and the use of bird-cliffs, the nearest of which is located only 1,200 m to the east of archaeological area D (Fig. 1). Pasture for the domestic animals and hunting grounds for reindeer and ptarmigan, along with archaeological evidence of wood, all suggest significant use of the inland hinterland of the site in the most recent past. The site has remained an important cemetery, meeting place, and sacred site for Samí culture right up to the present.

Pollen and spore data are also available from a mire within the archaeological site, (Fig. 1) (40) and they give a local signal of on-site activity. The pollen diagram (Supplementary Material Appendix Fig. S3) shows a typical vegetation history for eastern Varanger; pine is a dominant component until c. 1,700 cal. years BP when it falls below 20% TLP (Total Land Pollen), and after 500 cal. years BP it falls below 15% TLP, which is probably only the long-distance component of the pollen rain (15). The pollen diagram also supplements the archaeological phases outlined above, with the earliest clear anthropogenic signal being a rise in mugwort (*Artemisia*), ribwort plantain (*Plantago lanceolata*), and microcharcoal from 6,900 cal. years BP. The record of cultivars fits with the archaeology with rye (*Secale*) first appearing c. 1,320 cal. years BP (630 CE) and then again at 880 cal. years BP, whilst barley (*Hordeum*) appears at 660 cal. years BP.

### Nordvivatnet

**Fig. 2.**
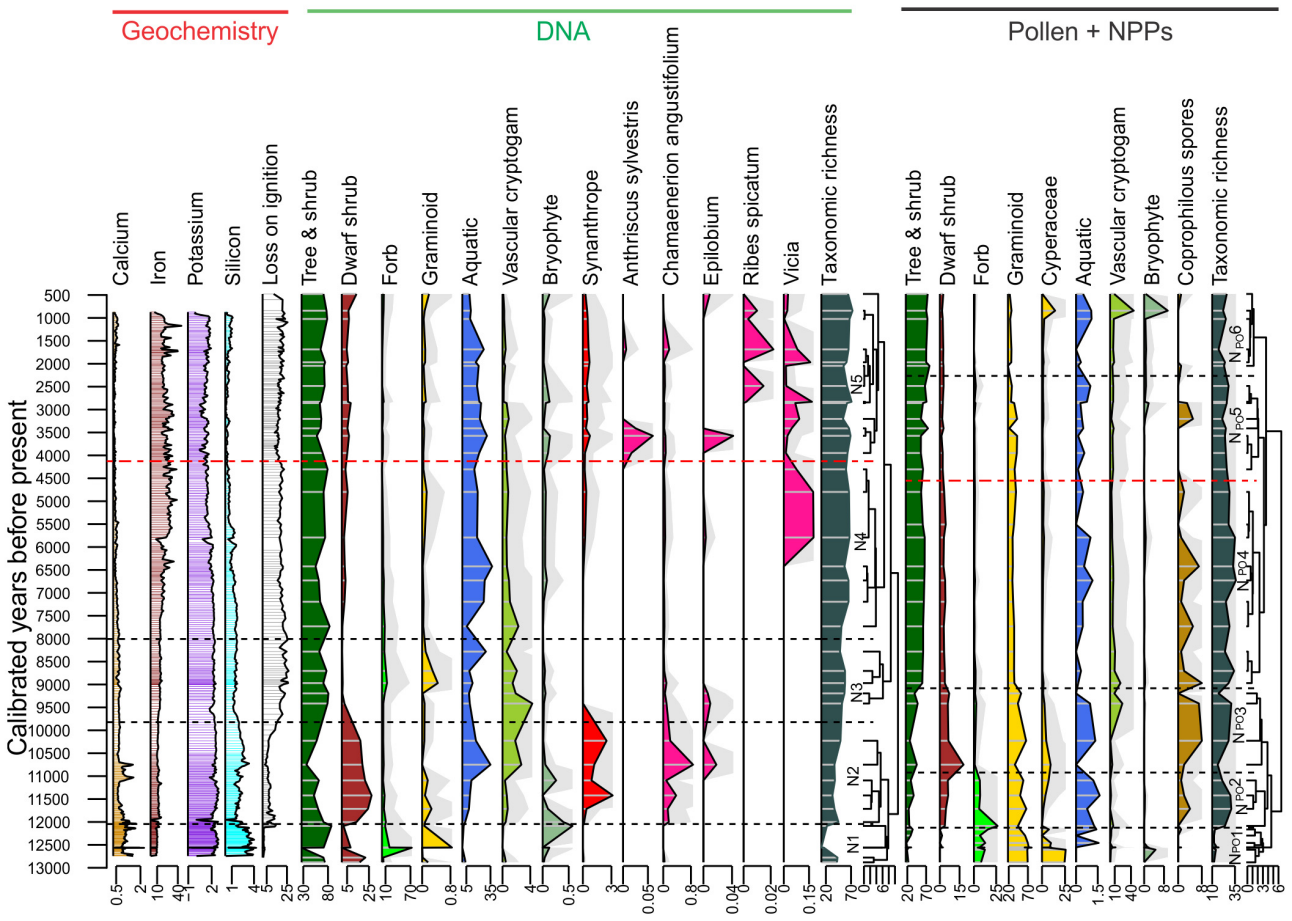
Nordvivatnet: selected XRF elements, LOI, sedaDNA-based plant functional groups based on proportion of reads divided into zones (N1 to N5a, b), and pollen proportion of functional groups divided into pollen assemblage zones (N_po_1 to N_po_6). Gray silhouettes are ×10. Note the variable scales (incl. ×10) and the high “synanthrope” (taxa associated with humans) values in the Early Holocene—see text for discussion. The vegetation zones with lower broken-stick support are indicated by double-dashed red lines. Graminoid in the pollen excludes Cyperaceae, which is shown separately.

Analysis of a 3.2 m sediment core from Lake Nordvivatnet (70.13305 N, 29.01195 E; Fig. 1) with a robust chronology (Supplementary Material Appendix Table S2 and Fig. S4) revealed a rise in organic matter from the later part of the Younger Dryas c. 12,000 cal. years BP, followed by a more gradual rise at c. 10,000 cal. years BP, with values remaining constant until a reduction at c. 4,000 BP and again at c. 600 cal. years BP (Fig. 2). At the base, the core revealed clastic sediments (clay-silts and stones) with some evidence of slumping interpreted as marine in origin. Above this basal unit, low and generally decreasing values for calcium and silicon are seen after 9,800 cal. years suggesting a fairly stable system (as does the accumulation rate) with increasing biological cycling (calcium, potassium, and silica). Iron, however, shows an increasing trend, particularly after 5,800 cal. years BP, which may reflect podzolization and peat formation (Fig. 2).

The plant sedaDNA metabarcoding of Nordvivatnet provided a total of 178 taxa (Supplementary Material Appendix Fig. S5a to d) and the vegetation time series has an average time-step of 350 years. The plant record is particularly rich and despite methodological problems associated with sedaDNA (45), can be regarded as reliable, although not perfect, due to the known stability of sedaDNA in saturated lake sediments (45). Our data is robust also because of an analytical combination of clean conditions, a conservative bioinformatic approach, and access to a full genome reference set for this region (PhyloNorway). Therefore, most errors are false negative rather than false positives (45, 46), although the correlation with other sites in the region with both sedaDNA and pollen data suggests that this is also rare (33). Using constrained incremental sum of square (CONISS) and a broken stick model, five statistical zones were identified (N1 to N5). For the pollen, a further subdivision of zone 5 was also made using the next statistically defined boundary below the broken stick inflection.

Zone N1 covers the Younger Dryas and is not considered further here due to it predating human occupation at this site, which at the time was a marine embayment. Zone N2 (Early Holocene 11,700 to 9,900 cal. years BP, Fig. 2) is dominated by the woody genera birch and willow (*Betula* and *Salix*) along with Arctic tundra shrubs (for full taxa, see Supplementary Material Appendix Fig. S5). A synanthrope peak identified during this period is due to pioneer species (see later discussion) and early Holocene warmth is indicated by the rise in aquatics. In zones N3 to N4 (9,700 to 4,100 cal. years BP), taxonomic richness remained stable (Fig. 2) and the local vegetation was dominated by trees (birch, mountain ash, and willows) and a range of shrubs. Pine (*Pinus*) also appears in this zone in both sedaDNA (and pollen) at levels that imply it was in the lake catchment (Supplementary Material Appendix Fig. S5a and 6a). In zone N5 (4,100 to 500 cal. years BP), there is little change in trees although pine decreases in the last 1,500 years. Forbs that include indicators of both climatic change (i.e. a reduction in temperatures) and human activity also appear in this zone as exemplified by *Bartsia alpina* (velvetbells) and *Ribes* (currant), respectively.

In the absence of cultivars (apochores) from the lake, human impact is assessed using a combined sum of facultatively synanthropic (apophyte, Supplementary Material Appendix Table S3) taxa known to be increased by human activity, including ruderal species and species that are increase by grazing by reindeer or domestic livestock. Synanthropes peak during the earliest Holocene, are then absent, reappear c. 6,500 cal. years BP but then peak in zone N5a to 5b at around 2,000 cal. years BP. This category includes cow parsley (*Anthriscus sylvestris*), willow-herb (*Epilobium*), peavine (*Lathyrus*), field forget-me-not (*Myosotis arvensi*s), common buttercup (*Ranunculus acris*), globeflower (*Trollius europaeus*), and bird vetch (*Viccia* cf. *cracca*). Several of these taxa, including willow-herb, are also found in the earliest Holocene as they are pioneer, r-selected strategists suited to open, and disturbed ground, which is common under both periglacial conditions and human disturbance (47).

Pollen and NPPs from Nordvivatnet indicate a broadly similar pattern of change in vegetation composition as the plant sedaDNA (Fig. 2; Supplementary Material Appendix Fig. S6a and b) although there are differences. Both show an increase in trees (mostly birch and pine) in the early Holocene along with a rise and peak in dwarf shrubs and a peak in forbs during the Younger Dryas–Holocene transition period. Vascular cryptograms show a similar pattern with a peak in the early Holocene. The differences include the earlier rise in pine in the pollen and a much higher representation of grasses (Graminoid) in the pollen both of which reflect the long-distance pollen component that is not present in the sedaDNA record. Other differences also reflect the different taxonomic resolution of the data (sedaDNA being better resolved), which also precludes a statistical comparison of the two records.

A rise in birch and willow pollen is simultaneous with a rise in forbs at the beginning of Zone N_po_2 c. 12,200 cal. years BP, indicating the regional establishment of an open willow and birch forest. This is likely to be open and probably low tree-birch forest rather than shrub-tundra (with dwarf birch) due to the similarity to values found in the Late Holocene when it is known that the lake was surrounded by birch-willow forest and other sites in northern Norway (33). Openness is indicated by pollen of dwarf ericaceous shrubs such as *Vaccinium* sp., crowberries (*Empetrum*) and heather (*Calluna*), which also increase at this time (Supplementary Material Appendix Fig. S6a). An increase in lake productivity is evident around c. 12,000 cal. years BP as indicated by the first appearance of pollen/NPPs from aquatic plants and algal taxa (*Potamogeton, Pediastrum* sp., *Botryococcus*). An increasing proportion of pine pollen is observed and remains high until the uppermost sample at c. 500 cal. years BP. Birch, pine, and the dwarf shrubs remain dominant throughout Zone N_po_3. There is a low number of facultatively synanthropic species, and they show no coherent increase over time. However, there are distinct peaks in coprophilous fungal spores with a rise in the early Holocene (N_po_3, c. 11,000 to 9,500 cal. years BP) during the period of birch-forest establishment with fluctuations in the middle Holocene (N_po_4) and a reduction in the late Holocene (N_po_5 to N_po_6) except for a spike at c. 3,400 cal. years BP, which is dominated by *Sporormiella* sp. which suggest phases of more intense grazing around the lake (Fig. 2; Supplementary Material Appendix Fig. S7). The earlier, more prolonged peak predates the pine rise (in both sedaDNA and pollen) and may reflect high reindeer grazing levels in the open birch forest to the west of the advancing pine forest front, which by 11,595 to 10,776 cal. years BP was at the head of the Varanger Fjord (14). Sea level at this time would also have been at the beach barrier so this was then a narrow coastal zone. Concentrations of microscopic charcoal are low throughout the sequence but highest in zones N_po_1 to N_po_2 (reaching 0.4 fragments cm^−2^yr^−1^) in the relatively open environment prior to human arrival due to natural fires. The microcharcoal which is supported by a peak in *Neurospora* (HdV 55—a genus of ascomycete carbonicolous fungus) is common in the Younger Dryas and early Holocene and its reduction in the remainder of the Holocene could be related to a reduction in natural fires due to lower summer temperatures and increase in precipitation (15).

**Fig. 3.**
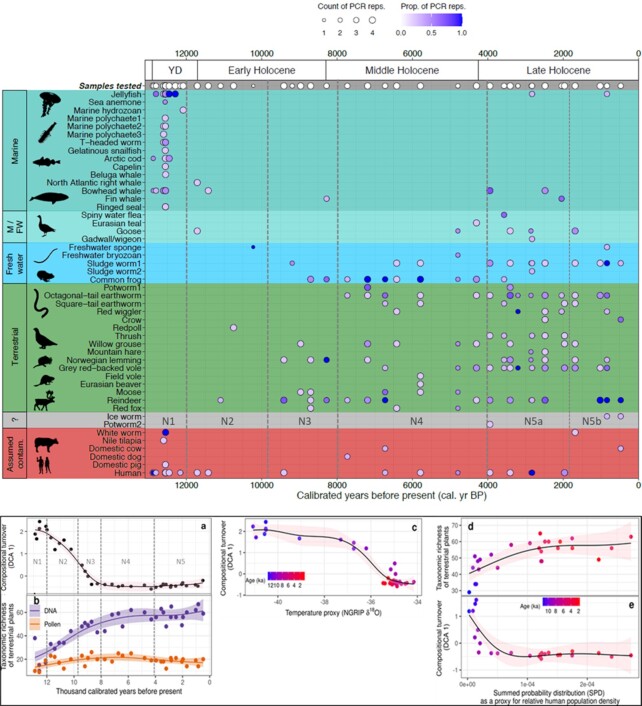
(a) Detection of animal sedaDNA from 45 samples throughout the Nordvivatnet record with sedaDNA plant zones. Background colors delineate habitat categories. The taxa with a gray background could not be assigned to a habitat category, whereas those with a red background are assumed contaminants. Full taxonomic information is in Dataset S2 and Dataset S3. The same data plotted by depth and including the negative controls are in the Supplementary Material Appendix Fig. S5. Silhouette credits are in the Supplementary Material Appendix Text (c). M/FW: Marine/Freshwater. (b) Temporal trends of terrestrial plant species (a) composition and (b) richness, and pattern of (c) species composition along a proxy for climate (NGRIP δ18O) and pattern of both the (d) richness and (e) composition along a proxy for relative human population density. All the fitted lines are based on generalized additive models and colored envelopes represent 95% CIs of estimates.

We also analyzed animal sedaDNA from Nordivivatnet using a primer set that was designed to amplify a short region of the mitochondrial 16S locus. Although originally designed for mammals, it has been previously observed that nonmammalian animal taxa, including invertebrates, can also be amplified using 16S primers (48). We exploited this “bycatch” to reconstruct the occurrences of 42 animal taxa, of which only 13 are mammals (Fig. 3a, Supplementary Material Appendix Fig. S8). Of these, 40 could be assigned to a broad habitat type. We note that detections of individual animal taxa are sporadic, limiting the accurate identification of first and last appearance dates, but combinations of taxa from our uniquely rich data set allow confident broad ecological interpretations. We only detected marine taxa in the Younger Dryas (plant sedaDNA zone N1, 13,000 to 12,000 cal. years BP), which is characterized by multiple occurrences of Lion’s mane jellyfish (*Cyanea* cf. *capillata*), suggesting that Nordvivatnet was a lagoon or beach-flat at this time prior to the development of the beach barrier that today confines the lake. This would explain the rapid deposition of sediments with low organic matter and glacially scoured stones (Supplementary Material Appendix Fig. S4), as well as the general poor quality of the plant sedaDNA results from zone N1. Within the earliest Holocene (zone N2, 12,000 to 9,800 cal. years BP), there is a sporadic and mixed assemblage, with a first occurrence of reindeer (*Rangifer tarandus*). Several marine taxa were detected with sporadic occurrences thereafter, which we explore in the Supplementary Material Appendix Text (a). From the latter half of the Early Holocene (zone N3 onwards), a variety of freshwater and terrestrial taxa, ranging from sponges to reindeer, characterize the animal sedaDNA data. Of note, the occurrences of common frog (*Rana temporaria*), moose (*Alces alces*), and red fox (*Vulpes vulpes*) from c. 9,000 to 3,500 cal. years ago, are consistent with a warmer than present climate and forest cover. Intriguingly, between c. 6,500 and 4,500, cold-adapted taxa of open habitats, such as reindeer and Norwegian lemming (*Lemmus lemmus*), drop out of the record and at c. 5,700 cal. years BP, we observe the only occurrence of Eurasian beaver (*Castor fiber*), a taxon that is absent from northern Norway today, suggesting warmth and forest cover were at their maxima over this interval. This occurrence of beaver also falls within the age range covered by the Old Stone Age house R12 from Mortensnes, which contained beaver bones (Supplementary Material Appendix Table S1). Of particular interest during the Late Holocene is the record of reindeer, which were absent during the Samí Iron Age (c. 2,500 to 1,000 cal. years BP) but are consistently detected with multiple haplotypes in the three uppermost samples (c. 1,000 to 500 cal. years BP), which cover the Samí Medieval period (Supplementary Material Dataset S3).

### Statistical testing

In order to test for a possible dependency of these ecological changes on climate and human influence, generalized additive modeling (GAM) was applied to the Nordvivatnet plant sedaDNA, pollen data, and summed probability distribution (SPD) of 14C dates from archaeological sites in northern Norway (8) (Fig. 3b). The plant composition as reflected by beta diversity was represented with the first axis scores from detrended correspondence analysis (DCA; based on weighted PCR replicates of terrestrial plants). The GAM analysis of plant composition against sample age shows a continuous decrease in species turnover from the Younger Dryas to 9,000 cal. years BP and a stable but low species turnover thereafter [Fig. 3b(a), Table S4]. Lower temperature tends to favor higher species turnover, which decreases gradually with increasing temperature. Richness increases in the early Holocene, plateaus in the mid Holocene in both sedaDNA and pollen but then rises again in the late Holocene in the plant sedaDNA data, but the same pattern is not reflected in the pollen-based data [Fig. 3b(b)]. The late Holocene rise is due to late arrivals into the catchment, many of which are related to human influence (synanthropes) rather than falling temperatures as suggested by the GAM analysis with NGRIP d^18^O, a proxy of paleotemperature [Fig. 3b(c)]. The plant richness increases with SPD values until ca. 7,200 then remains stable for rest of the record, despite the increase in human population [Fig. 3b(d)]. The beta diversity decreases rapidly from ca. 11,400 to ca. 9,000 and then remains insensitive to human population density [Fig. 3b(e)].

## Discussion

### SedaDNA, pollen, and archaeological data

**Fig. 4.**
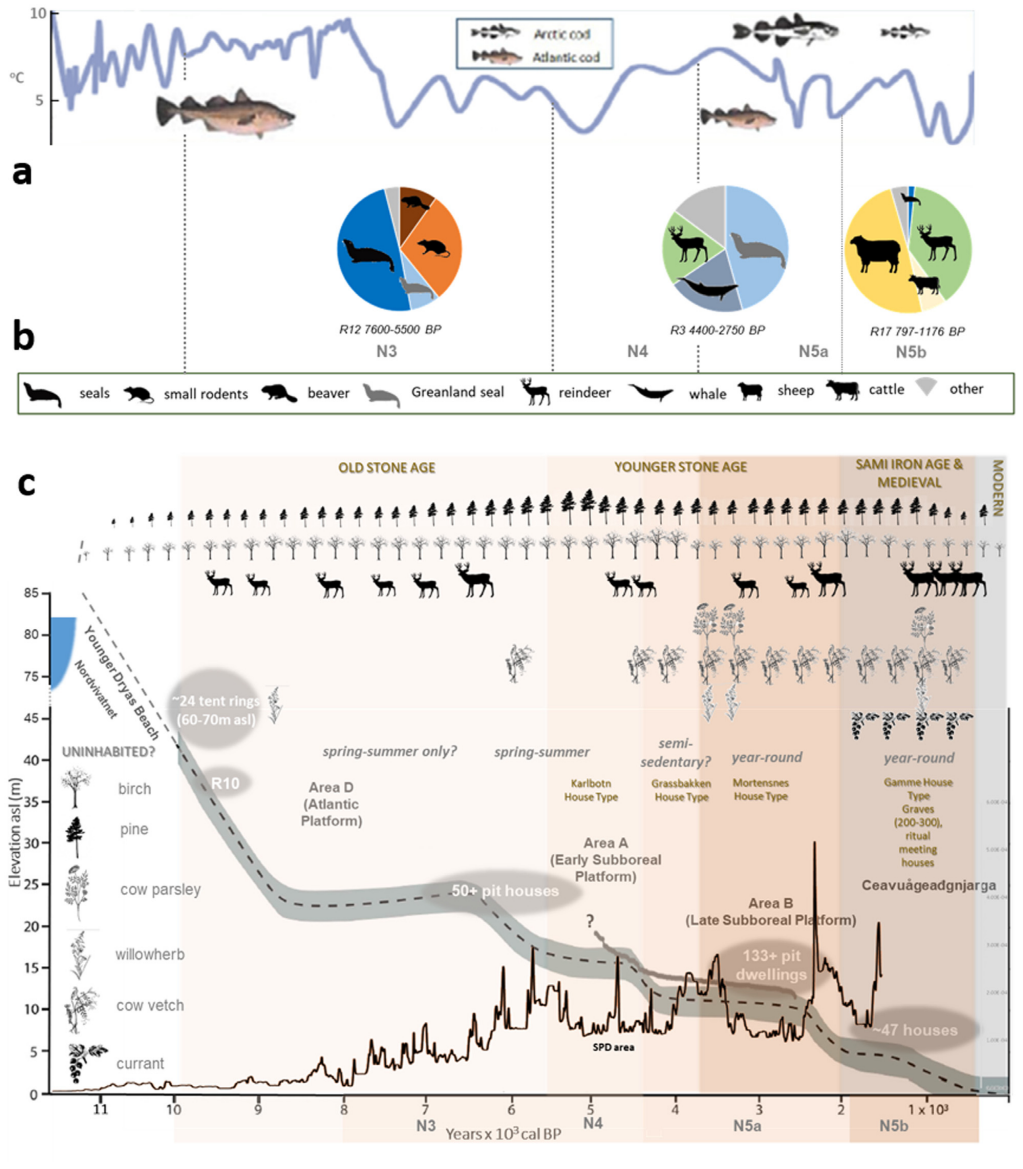
(a) Barents Sea margin sea temperatures inferred from foraminifera from ref. (50) with typical Arctic fish (Arctic cod) and Atlantic fish (Atlantic cod). (b) The summary of faunal data from three houses, (c) the summary of faunal data from three houses at Mortensnes with their date ranges calculated from ref. (35) and Supplementary Material Appendix Table S1, (c) Mortensnes archaeology superimposed on the Varanger Fjord relative mean sea level curve (msl) from ref. (51) with the house height data from ref. (36) (gray circles) adjusted in age to fit with the msl curve (broken line) and a summary of key faunal data from Nordvivatnet and the SPD area curve from ref. (8) for northern Norway. The size of the reindeer icons reflects the number of sedaDNA repeats (0 to 4).

By combining the data, we can identify four Holocene phases (N3 to N5) superimposed on the five traditional archaeological phases, that show different ecological impacts around Lake Nordvivatnet and the Mortensnes archaeological site (Fig. 4). In the first Holocene phase (N3, 9,900 to 8,000 cal. years BP), which equates to the Early Holocene and pioneer/Old Stone Age phase at Mortensnes, there is no evidence of human impact from any data. Reindeer is recorded sporadically in the sedaDNA, and grazing probably occurred around the lake as recorded by the fluctuating coprophilous spores values (Fig. 2, Fig. S7). In the second phase, which is the middle Holocene (8,000 to 4,200 cal. years BP, N4), there is evidence of beaver at Nordvivatnet and of predominantly marine mammal hunting from the site itself, along with a rise in on-site apophytes after 6,800 cal. years BP. In the third phase, the early Late Holocene (4,200 to 2,000 cal. years BP, N5a), the presence of synanthropic species reflects grazing at Nordvivatnet, and there is evidence of hunting of sea mammals (particularly seals) and reindeer from the site. In the last recorded phase, the later Late Holocene (2,000 to 500 cal. years BP, N5b), there are stronger indications of human activity around the lake, including a reduction in tree cover, the appearance of currant bushes (*Ribes*), and strong evidence of reindeer. In this period, the on-site evidence is dominated by reindeer with domesticates, principally sheep, and cereal cultivation. Mortensnes records the second-most northerly, and the most easterly, occurrence of cereal pollen in Fennoscandia; the only more northerly occurrence is from Risfjorddalen, Melham (71°N), which is dated to 2,450 and 2,800 cal. years BP (49). At Mortensnes, the occurrence of arable domesticates also agrees with the archaeological evidence of domesticated stock in the Samí Iron Age (<2,000 cal. years BP).

### Archaeology, population, and culture

Combining the data, it is clear that the economic character of Mortensnes changed over time, from one dominated by the use of the sea and coast in the Older Stone Age to a more mixed economy using more parts of the landscape in the Younger Stone Age. This may reflect a change in hunting pattern, with early hunting occurring further inland, but also it signals a trend towards sedentism, which can also be argued from the archaeology (35). Finally, in the last thousand years, there developed an economy that was partly agricultural, along with the continued use of reindeer (probably herding) and selected coastal resources, including bird cliffs. This does not reflect the density of structures over time, which suggest that the Younger Stone Age and Early Metal Age (c. 7,000 to 2,000 cal. years BP) had a higher density of occupation than the last millennium. A time proxy that can augment the radiocarbon dates is the height above sea level, and this shows a peak in house density at 8 to 12 m asl. While it is the case that not all houses would have been occupied simultaneously, this does suggest population growth during the broad time period of 4,400 to 2,000 cal. years BP (Gressbakken and Mortensnes Phases, Fig. 4). These data can also be compared with the regional SPD analysis of archaeological radiocarbon dates for northern Norway that has been interpreted as 3 to 4 boom-bust cycles (8), and which can also be regarded as waves of inward migration. This shows a rise in the pioneer phase to a peak at c. 6,000 cal. years BP (Mesolithic migration possibly from both east and west), then relative stability until a peak c. 3,800 cal. years BP and another c. 2,800 cal. years BP, followed by a decline until a rise c. 1,800 to 1,500 cal. years BP. Although the SPD record ends at 1,500 cal. years BP, the house record from below 6 m asl suggests lower population densities than in the earlier periods and/or a change in the character of site use. This contrasts with the sedaDNA record of plant richness, reindeer density, pollen, and coprophilous spores (Supplementary Material Appendix Fig. S7), which reveal subtle evidence of grazing in the Younger Stone age but the strongest impact related to the introduction of domesticates, and probably reindeer herding, in the last 1,000 years (later Sami Iron Age and Sami Medieval Period).

In trying to interpret this temporal pattern, it is pertinent to return to the location and marine environment of Mortensnes. The importance of the Atlantic Boreal marine species increases with warmer sea temperatures and thus they became dominant in the Older Stone Age; this is happening again today with the warming of the Barents Sea (52). Varanger was ideal for skin-frame boats, making fishing both relatively safe and highly productive, although this appears to have declined in the later Holocene. The correlation of the SPD and marine temperature data found by ref. (8) is also consistent with the North Cape current being a driver of population dynamics before 1,500 cal. years BP. It is also significant that the interior, particularly the Varanger peninsula, has no settlement today but has evidence of some human use including early Metal Age Sites, reindeer hunting traps, seasonal herding camps, and corrals (53). The on-site zooarchaeological data suggest that marine and coastal resources were the early pull-factors for the region, with the addition of terrestrial subsistence resources, including furs, particularly late in the historical period, when the site was a regionally important Samí gathering site, a Norwegian trade post, and a farmstead with pastoral agriculture.

Using the most comprehensive multiproxy paleoecological record in the Arctic to date (sedaDNA of plants and animals, pollen, NPPs, microcharcoal, and faunal remains), we show that a site with a nearly complete Holocene record of human occupation had little or no discernable effects on its hinterland until the last 3,600 years, when grazing and probably wood-cutting is evidenced but was still low. It is only in the last 2,000 years that these effects increased and in the last millennium that we get continuous and sedaDNA reads of reindeer, suggesting herding. The plant record is shown here to diverge from climatic control in the last 2,000 years, and this is ascribed to human impact. Comparison with the archaeological data suggests that it is the changing economy associated with in-migration, from marine-orientated pioneers (from west and/or east), to mixed fishing (particularly sealing) and reindeer hunting, and finally terrestrially dominated pastoral agriculture in the Sami Iron Age and historical period, that has driven this record, rather than population per se. This implies that it is what people do, and probably where they came from, that drives environmental impact in this Arctic region, both in the past and probably into the future.

## Methods summary

Only a brief summary of the methods is given here and for more detail see the Supplementary Material Appendix Text b and the Supplementary Material Dataset S4. The lake site, Nordvivatnet, is a small lake perched behind a beach barrier 75 m above the site. It provides the nearest freshwater and is also on the easiest route to the north and into the interior of the Varanger peninsula. Its sediments were cored in 2017 and the lake surround surveyed for vegetation composition. We split the core, which was scanned using XRF and magnetic susceptibility. Plant sedaDNA was determined using methods described in ref. (33). We generated the animal sedaDNA data set from the same DNA extracts using the MamP007 primers to amplify a 60 to 84 bp fragment of the mitochondrial 16S locus. Pollen and spore analyses used standard extraction methods and reference material for both the Nordvivatnet and on-site sequences. Statistical methods included CONISS for vegetation zonation and GAM for modeling diversity patterns (Supplementary Material Appendix Fig. S9).

## Funding

The study is part of the ECOGEN project “Ecosystem change and species persistence over time: A genome-based approach,” financed by Research Council of Norway grant 250963/F20. The publication charges for this article have been partially funded by a grant from the publication fund of UiT The Arctic University of Norway.

## Supplementary Material

pgac209_Supplemental_Files

## Data Availability

All data are included in the manuscript and Supplementary data. The Holocene plant sedaDNA has been deposited in the European Nucleotide Archive (ENA) at project accession PRJEB39329, with sample accessions ERS4812035 to ERS4812048. Prefiltered ObiTools output files have been uploaded to figshare (DOI: 10.6084/m9.figshare.c.5468139) project accession code (PRJEB39329) as in ref. (33) and (Supplementary Material 56). The animal sedaDNA data have been uploaded to ENA (accession code is PRJEB56158). The raw pollen and NPP data are given in the uploaded Dataset File S10 to S12.
