# Correction to: Paleoeconomy more than demography determined prehistoric human impact in Arctic Norway

**DOI:** 10.1093/pnasnexus/pgad040

**Published:** 2023-02-15

This is a correction to: Tony Brown, Dilli P Rijal, Peter D Heintzman, Charlotte L Clarke, Hans-Peter Blankholm, Helge I Høeg, Youri Lammers, Kari Anne Bråthen, Mary Edwards, Inger G Alsos, Paleoeconomy more than demography determined prehistoric human impact in Arctic Norway, *PNAS Nexus*, Volume 1, Issue 5, November 2022, pgac209, https://doi.org/10.1093/pnasnexus/pgac209

In the originally published version of this manuscript, the sentence “Løkvika is dated by five radiocarbon dates to between 9,920 and 9,640 uncal. years BP and Cåkki is dated to 9,780 uncal. years BP” was incorrectly written as “Løkvika is dated by five radiocarbon dates to between 9,920 and 9,640 cal. years BP and Cåkki is dated to 9,780 cal. years BP.”

The following funding information was also erroneously omitted: “The study is part of the ECOGEN project “Ecosystem change and species persistence over time: A genome-based approach,” financed by Research Council of Norway grant 250963/F20. The publication charges for this article have been partially funded by a grant from the publication fund of UiT The Arctic University of Norway.”

Hans Peter Blankholm's name was incorrectly written as Hans-Peter Blankholm.

These errors have now been corrected.

