# Supplementary material for: Paleoeconomy more than demography determined prehistoric human impact in Arctic Norway
Source: PNAS Nexus. 2022 Oct 7;1(5):pgac209. doi: 10.1093/pnasnexus/pgac209 (PMC9802259; doi:10.1093/pnasnexus/pgac209)

**Supplementary Information: Figures Fig. S1-S9**

Fig. S1. Panel of photographs from Mortensnes. **a** Row of Younger Stone Age semi-subterranean pit-dwellings from Area D (R 16 in K. Schancke 1988) facing east. Copyright H.P. Blankholm. **b** Younger Stone Age Gressbakken type semi-subterranean pit-dwelling from Area A (R 3 in K. Schancke 1988) facing south. Copyright H.P. Blankholm. **c** Standing stone in Area B facing North-east. Copyright H.P. Blankholm. **d** Cemetery east of Area D (R5 in Schanche 1988) facing south. Copyright H.P. Blankholm.


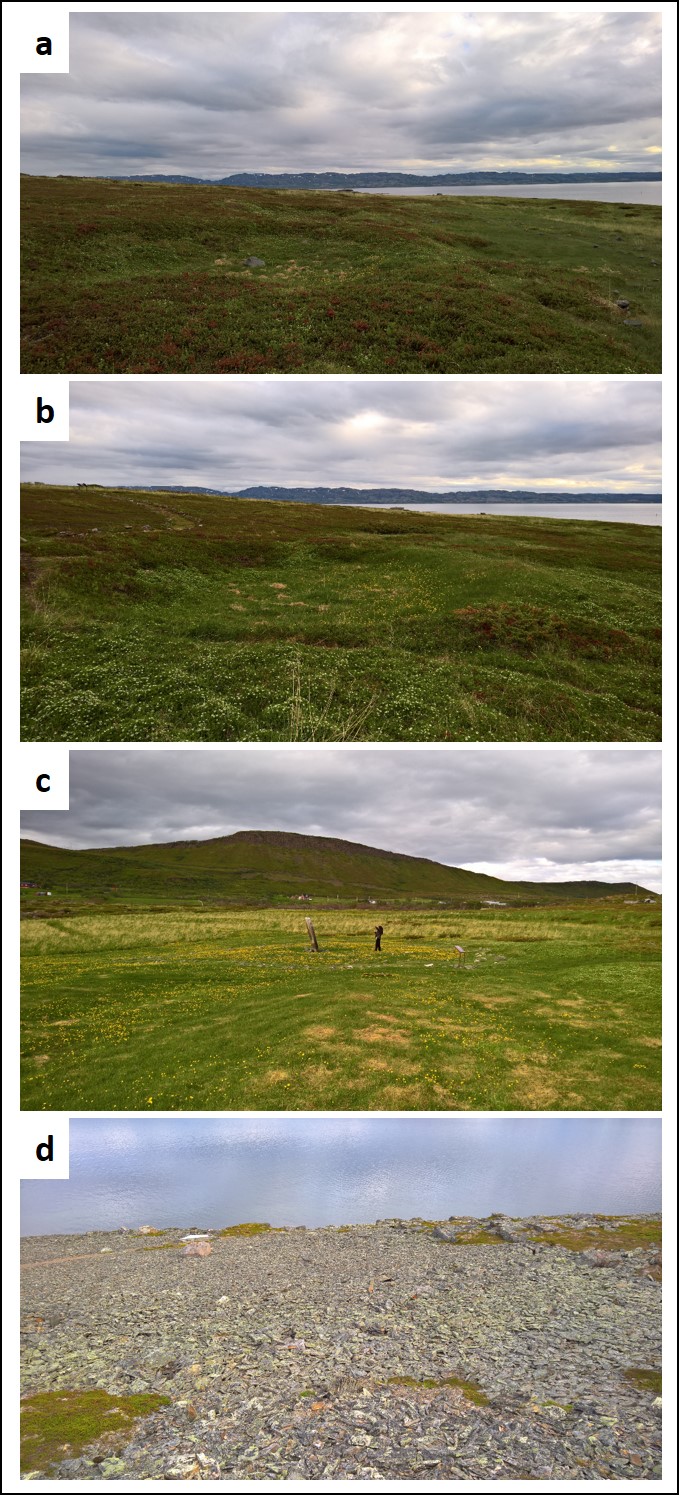


Fig. S2 The faunal data from houses R12, R3 and R17 recalculated from Schanche 1988 by both fragment number and weight (g). Pie charts are based on weight.


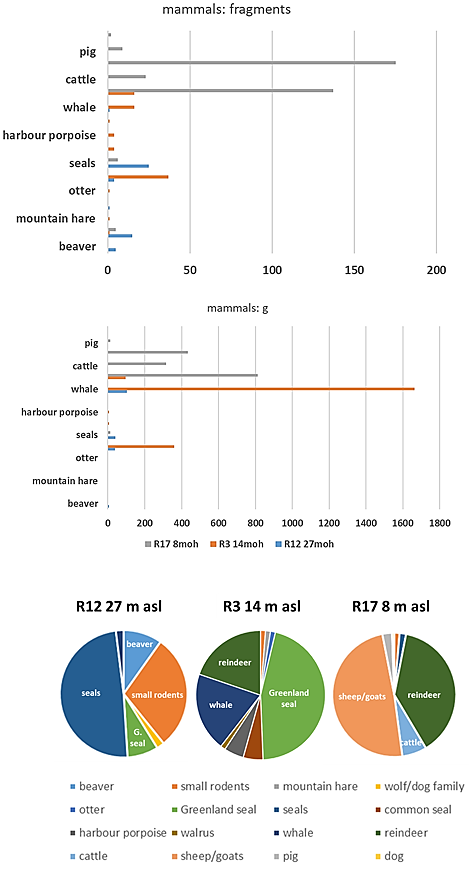


Fig. S3. Pollen diagram of selected taxa from the Mortensnes mire adapted and replotted from original data (43) as part of this research.


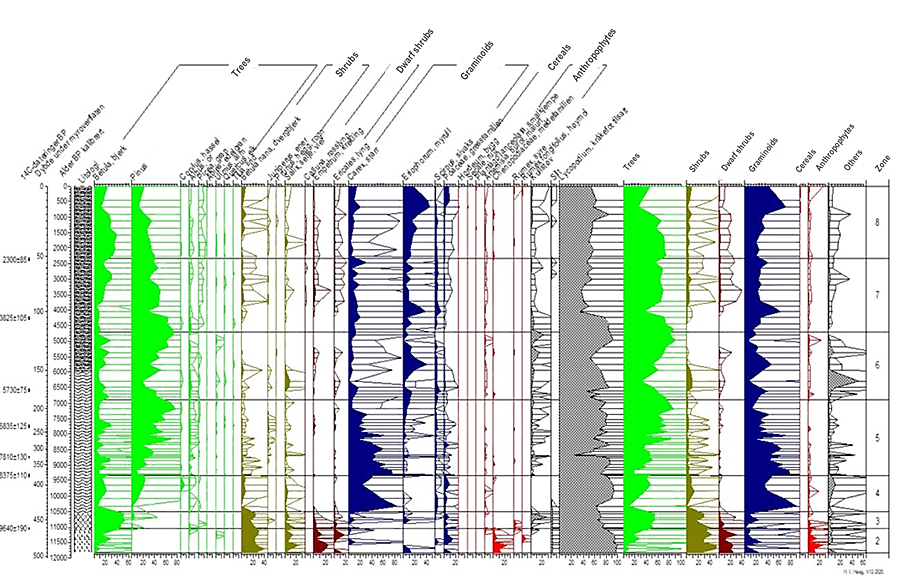


Fig. S4 The Nordvivatnet age-depth model generated in bacon and high-resolution imagery of the core, with The Younger Dryas sediments enlarged. Within the enlarged image, the concavities are from where glacially scoured stones were recovered. The age-depth model was originally published in Rijal et al. 2021.


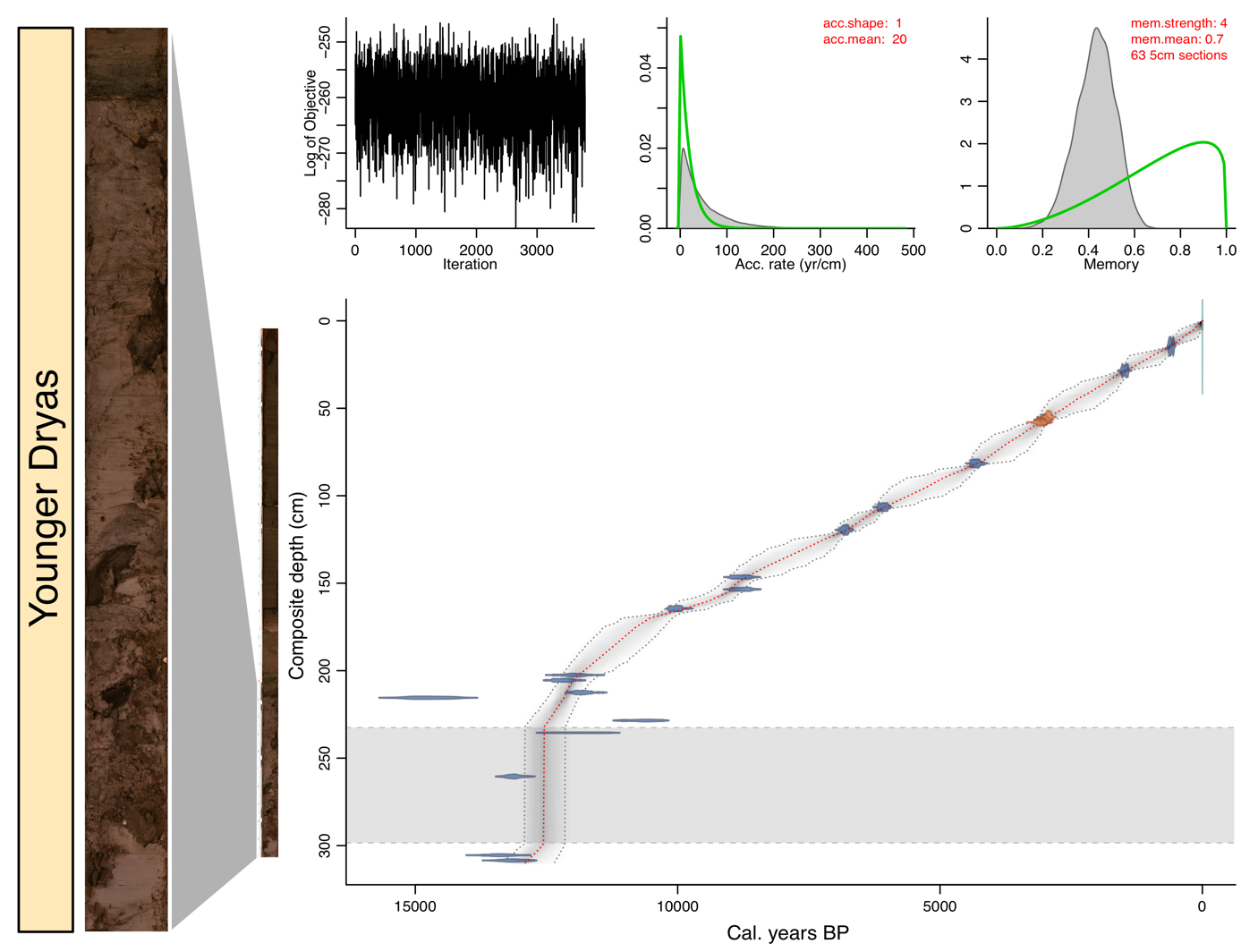


Figs. S5a-d SedaDNA data expressed as number of PCR replicates (1-8). List of plant functional groups: trees (mid-brown), shrubs (green), dwarf-shrub (brown), graminoid (yellow), aquatic macrophytes (blue) based on *seda*DNA from Nordvivatnet. Vegetation zones are based on constrained incremental sum of square (CONISS) analysis of all terrestrial plants.


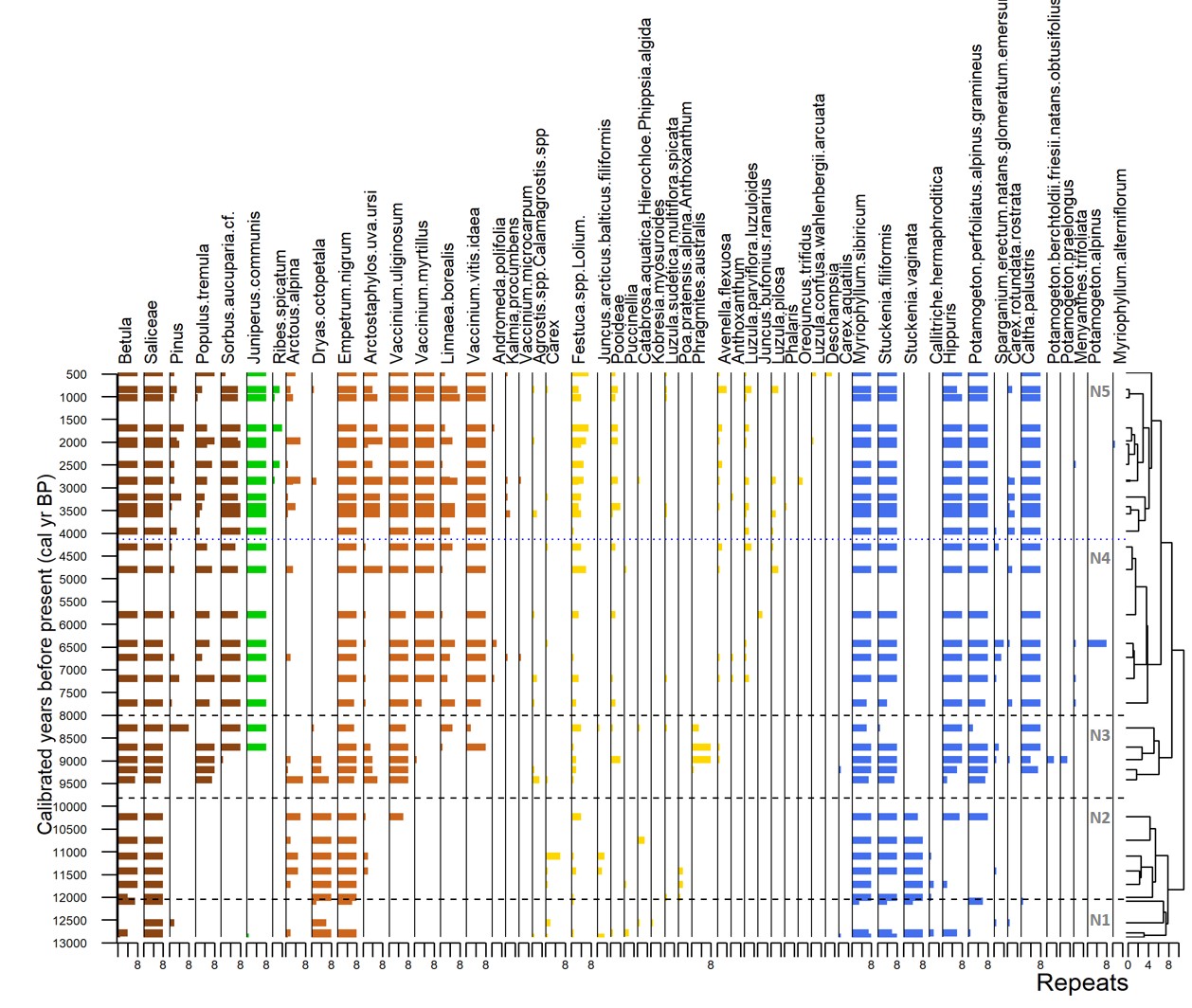


Fig. S5b Forbs


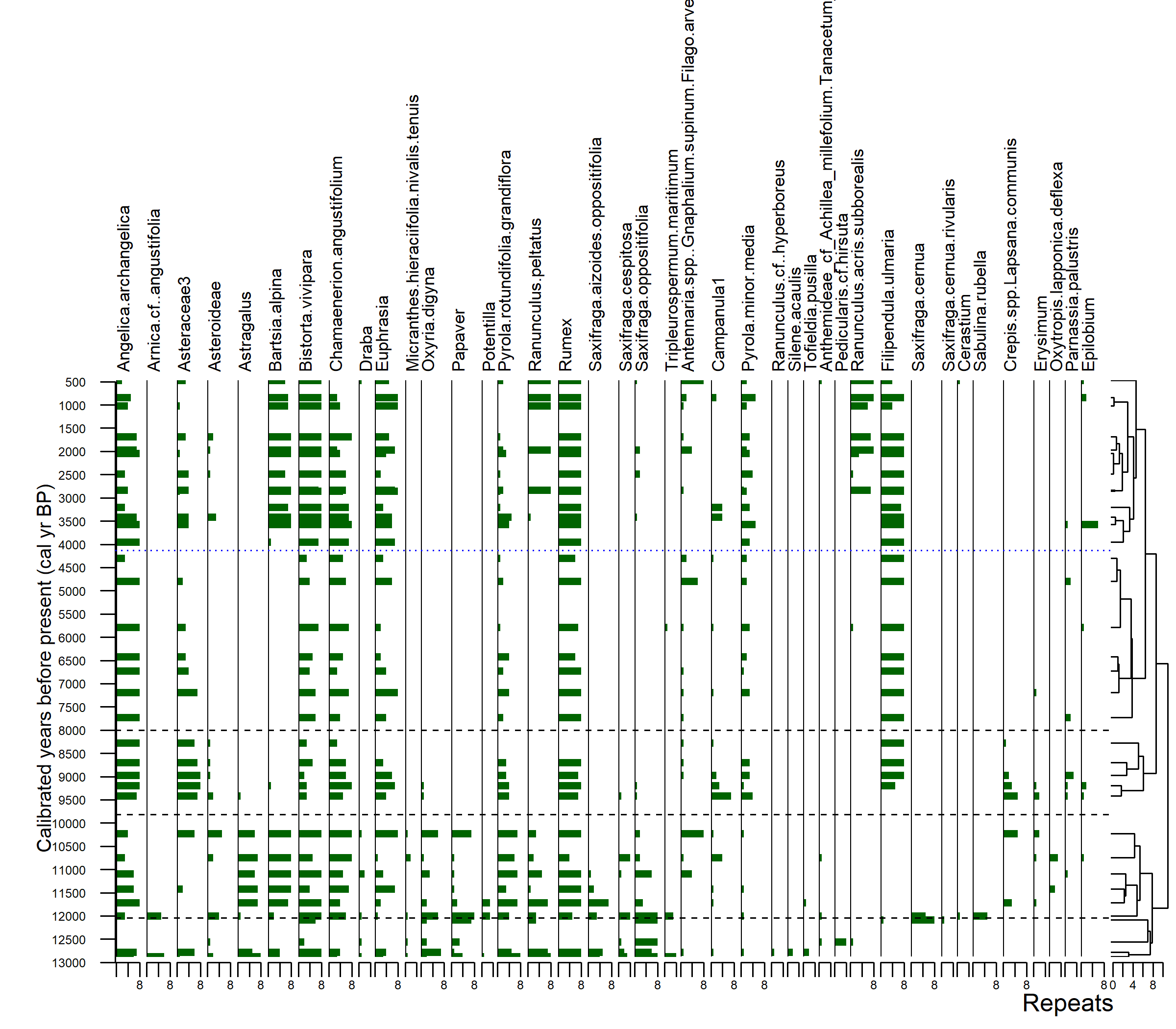


Fig. S5c Forbs (cont.)


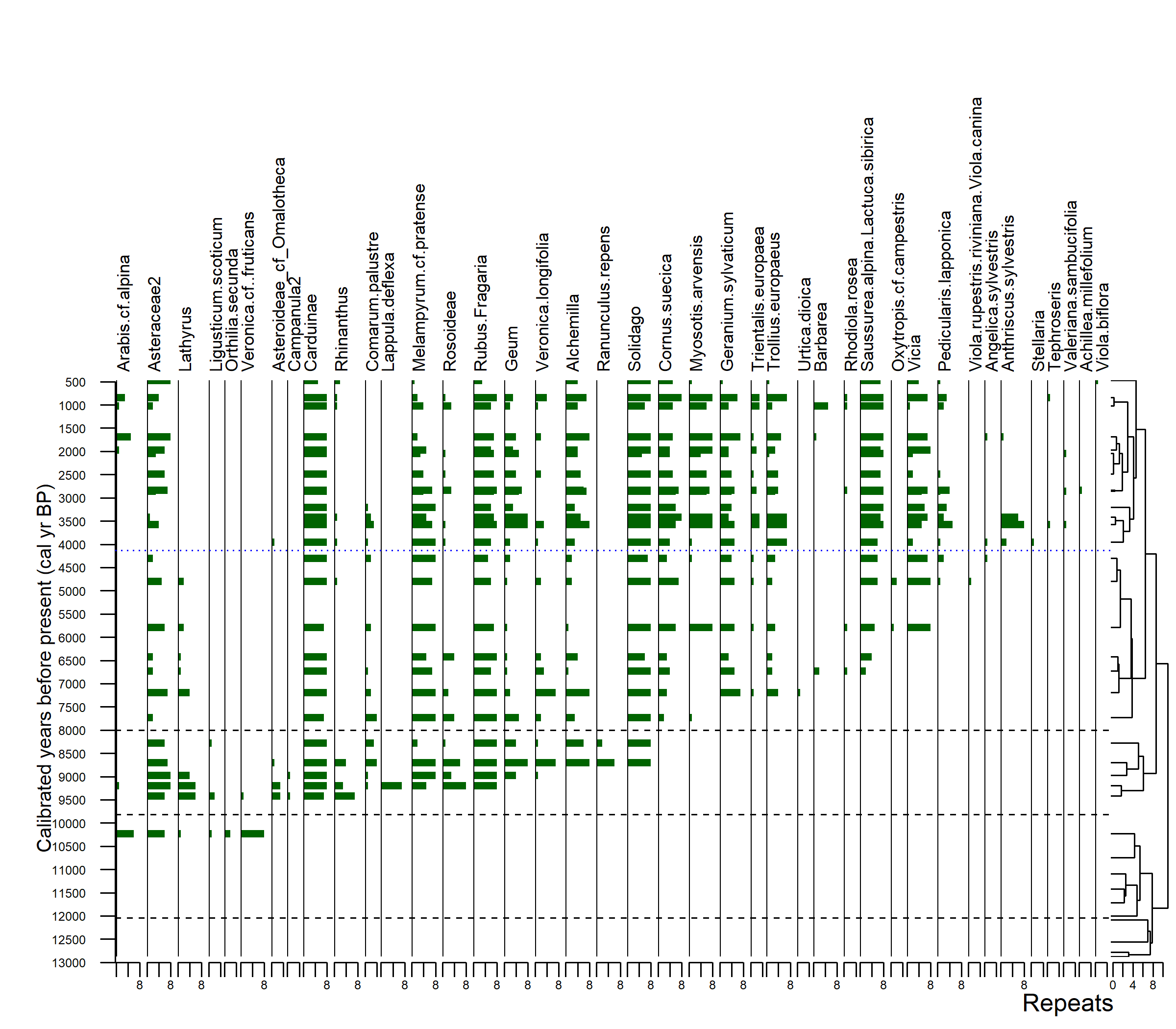


Fig. S5d Cryptogams: vascular cryptogams (light green), bryophytes (grey), algae (dark green)


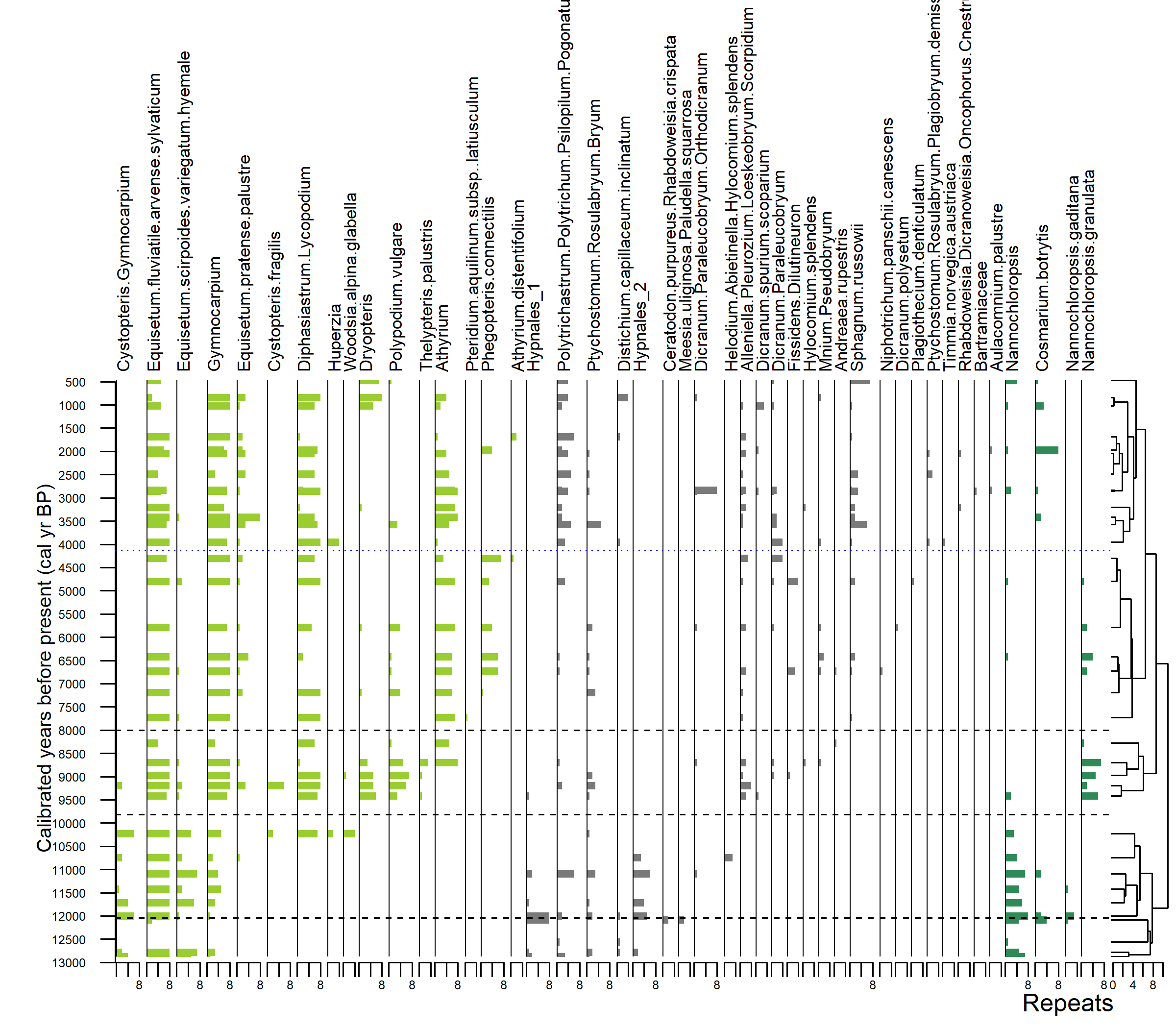


Fig S6a-b Plant functional groups identified in pollen analysis from Nordvivatnet: trees (dark brown), shrubs & dwarf-shrubs (orange), forbs (green), graminoids (yellow).


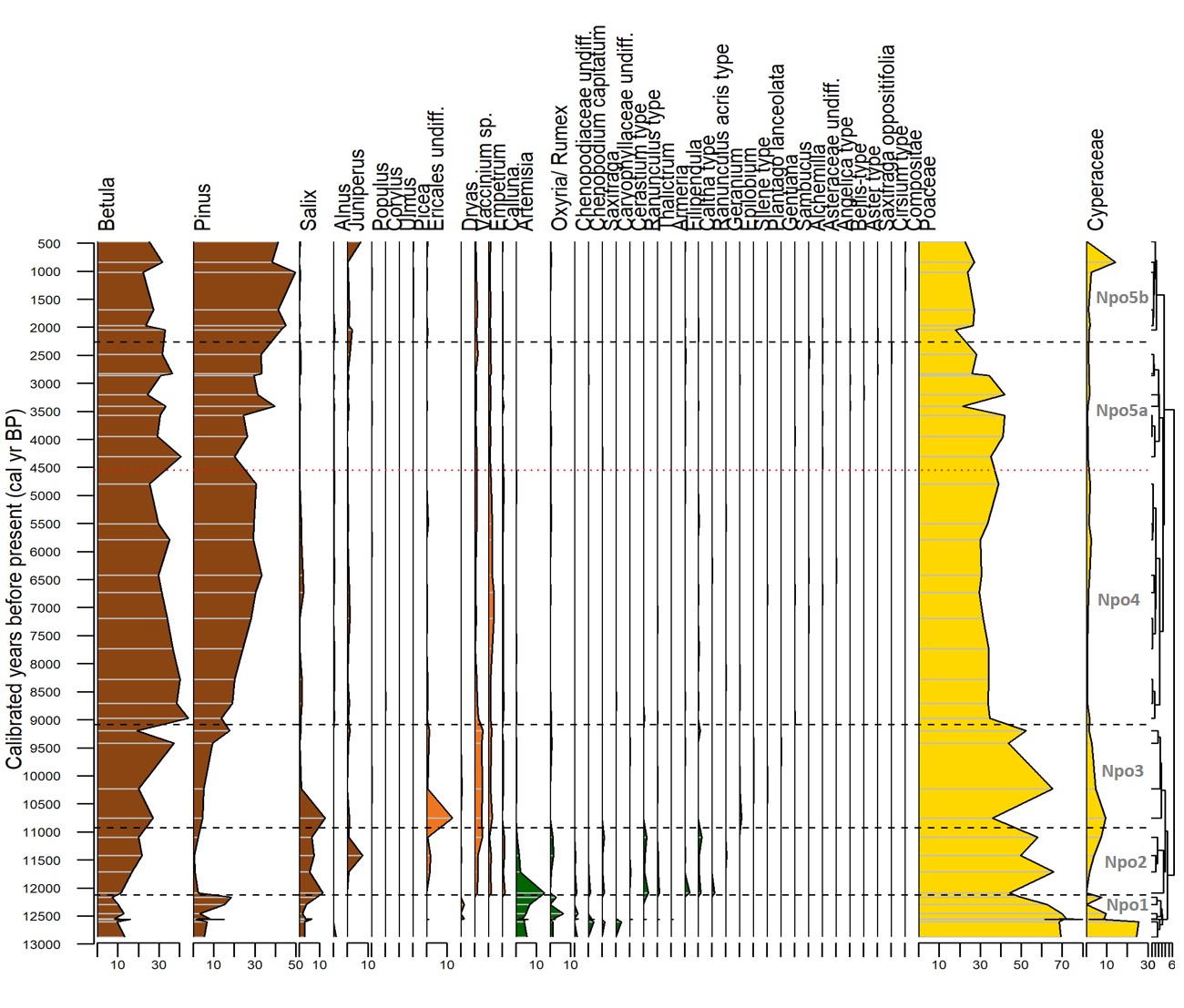


Fig. S6b Cryptogams identified in pollen analysis from Nordvivatnet: vascular cryptogam (pale-green), Sphagnum (grey), aquatic macrophytes (blue), algae (light green).


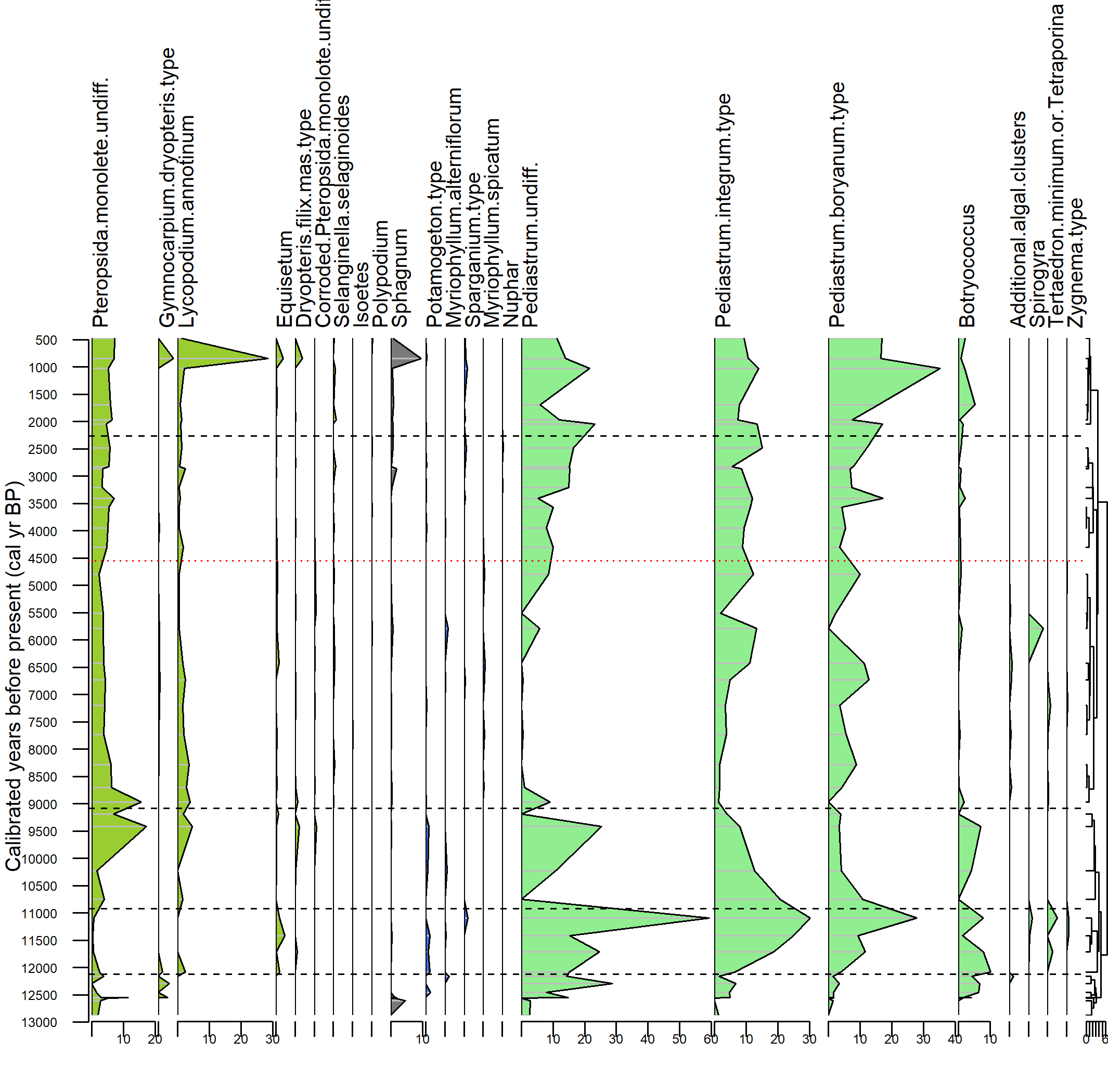


Fig. S7 NPPs from Nordvivatnet with HdV numbers where appropriate. Mycorrhizal fungi of Glomeromycota - *Glomus* (HdV 207, soil erosion indicator, olive brown), coprophilous spore types (mid-brown), plant associated including rusts (green), other fungal (grey) and algal spores (bright green), invertebrate remains (pink) and other miscellaneous remains (purple). Zones are the pollen assemblage zones.


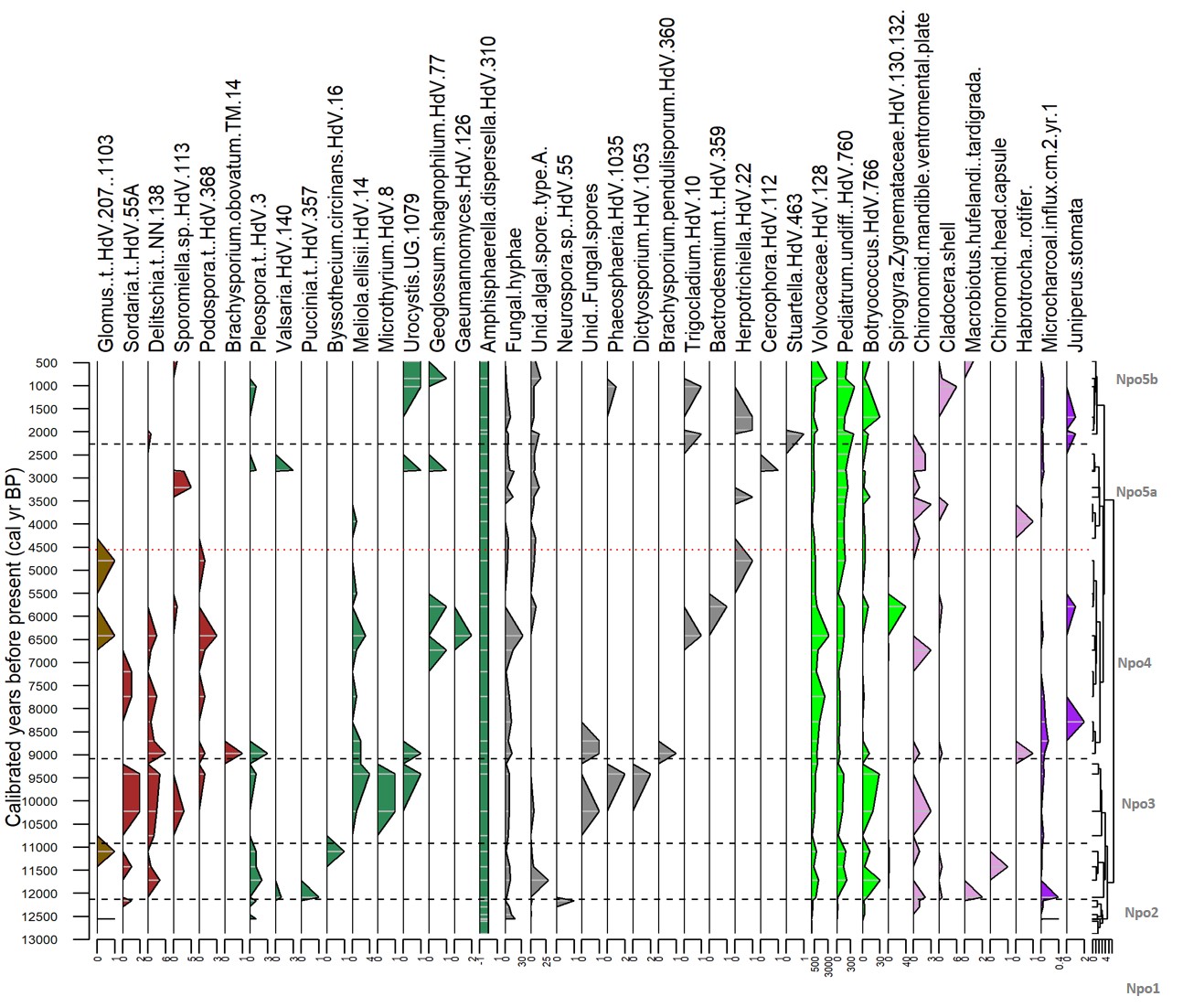


Fig. S8 Detection of animal sedaDNA from 45 samples throughout the Nordvivatnet record by composite depth. Background colors follow habitat categories. The taxa with a grey background could not be assigned to a habitat category, whereas those with a red background are assumed contaminants. Vertical dashed lines are CONISS zones, as defined by the plant sedaDNA data, with black lines indicating statistically significant zones and the grey line indicating the next statistical division. Full taxonomic information is in SI Appendix Dataset S2. Fig. 4 is the same data plotted by age. Silhouette credits are in the SI Appendix text. M/FW: Marine/Freshwater.


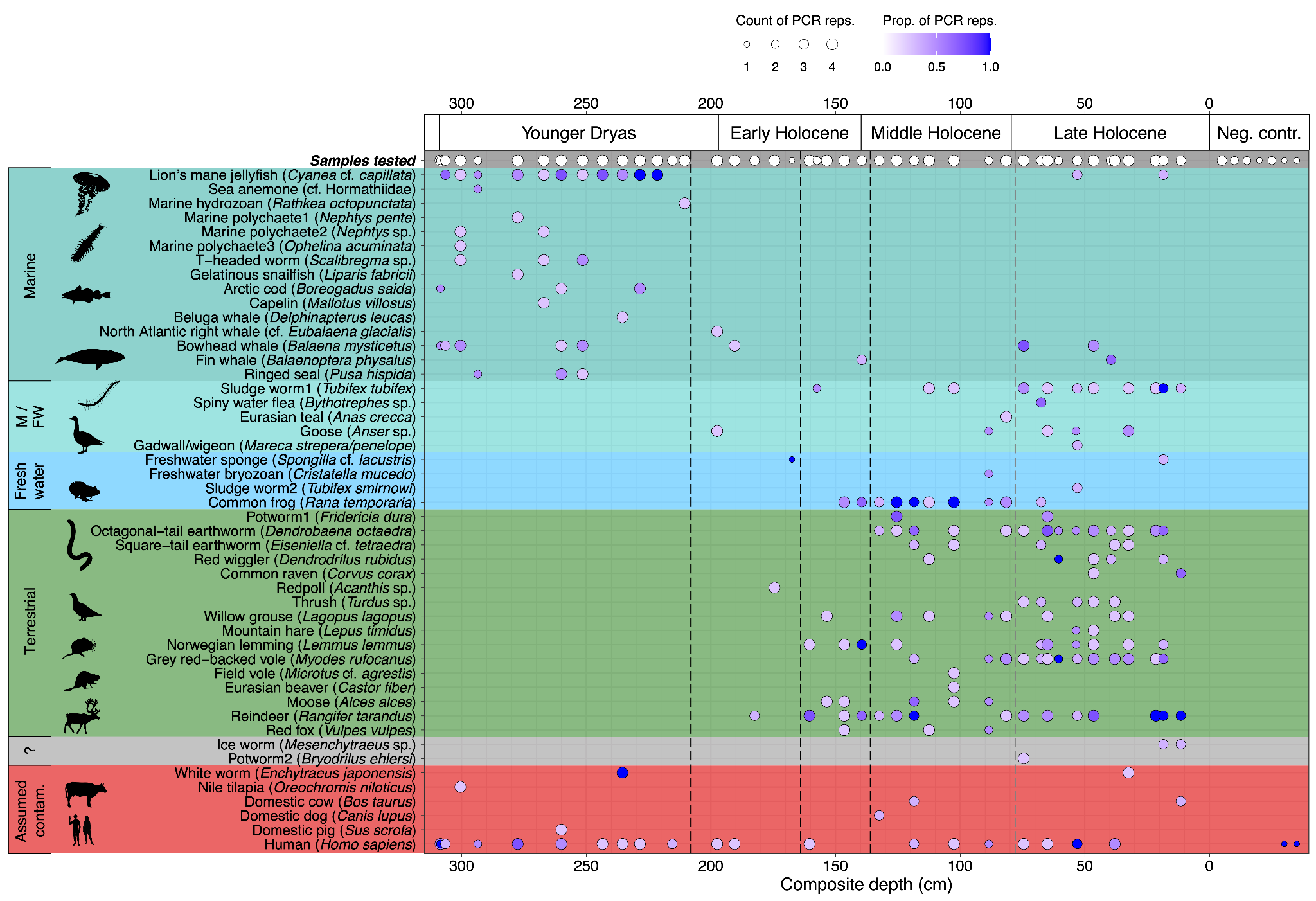


Fig. S9 Comparison between GAM and GAM-CAR1 models of compositional change through time. The first axis scores of detrended correspondence analysis (DCA) is considered as a measure of compositional change. The fitted lines and their 95% confidence intervals are based on either a generalized additive model (GAM) or a generalized additive model with a continuous time first-order autoregressive process (GAM-CAR1).


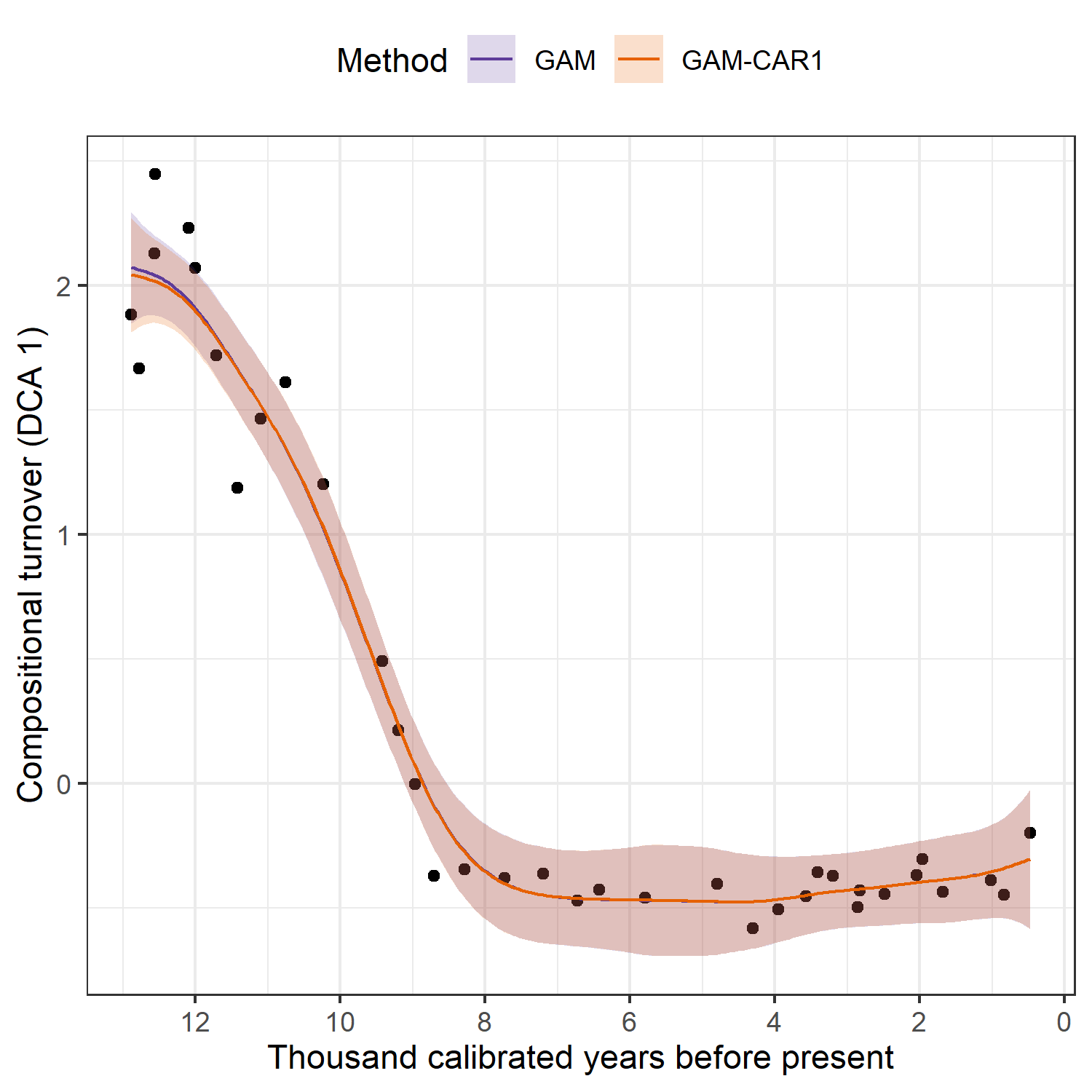

Supplement: pgac209_Supplemental_Files [file pgac209_supplemental_files.zip › PNASNEXUS-PNASNEXUS-2022-00238-T-s02.docx]
