# Supplementary material for: Paleoeconomy more than demography determined prehistoric human impact in Arctic Norway
Source: PNAS Nexus. 2022 Oct 7;1(5):pgac209. doi: 10.1093/pnasnexus/pgac209 (PMC9802259; doi:10.1093/pnasnexus/pgac209)
Supplement: pgac209_Supplemental_Files [file pgac209_supplemental_files.zip › PNASNEXUS-PNASNEXUS-2022-00238-T-s03.docx]

**Supplementary Information Appendix: Text and Tables**

**Text (a). A lingering marine animal sedaDNA signal in the Holocene**

We note that there is a lingering signal of marine animal taxa, consisting of cetaceans and jellyfish, in multiple samples across the entire Holocene (Fig. 3; SI Appendix, Fig. S6). We do not consider these observations to be a technical artifact for the following reasons: (1) all marine taxa are absent from the negative controls; (2) fin whale is not detected in the Younger Dryas samples, as would be expected in a cross-contamination scenario; (3) read counts are too high (up to 433) to preclude index-hopping artifacts from other sequencing libraries; (4) these detections occur in samples with a diversity of terrestrial and freshwater taxa and so are unlikely to have been the result of trace contamination; and (5) terrestrial taxa are entirely absent from the Younger Dryas samples, which would not be expected under an experiment-wide cross-contamination scenario.

We therefore propose two taphonomic hypotheses as to the origin of this lingering marine signal:

1. *Sediment redeposition*, which posits that the erosion of Younger Dryas sediments in the Nordvivatnet catchment resulted in the redeposition of older marine animal sedaDNA into the Holocene sediments.
2. *Sea-spray environmental DNA*, which posits that the marine animal sedaDNA is contemporaneous with the layers from which the DNA was sampled. A mechanism for this hypothesis is that Nordvivatnet is located near the coast with climatic conditions amenable to both sea-spray generation and DNA survival within sea-spray. Consistent with this hypothesis is that the two marine animal taxa detected in the Holocene sediments have high biomass; cetaceans, which also directly produce sea-spray through spouting, and jellyfish, which can reach massive biomasses during blooms.

Future work could test these hypotheses by further replication to test the presence (expectation of hypothesis 1) or absence (expectation of hypothesis 2) of non-cetacean and non-jellyfish taxa. Further, the age of the marine animal sedaDNA from the Holocene layers could potentially be assessed using the relative extent of deamination-induced DNA damage patterns, which is a time-dependent process.

**Text (b). Methods**

**Site, sediment coring and lithology.** Nordvivatnet (70.13565 N, 29.01195003 E) is a small-medium triangular-shaped lake (4.6 ha) at the southern edge of the Varanger plateau at 82 m a.s.l. overlooking the coast and the archaeological site of Mortensnes (Samí: Ceavccageađgi). It is dammed by the highest raised beach on the peninsula with a beach-ridge sill at 85.8 m asl. The catchment area is only 0.2 km^2^, just extending to the peaks of the slopes that surround the lake. The lake is the nearest freshwater supply to the archaeological site, albeit being perched about 55m above the site and around 50m above the coastal zone. The bedrock underlying the catchment is conglomerate of the Mortensnes formation, which is matrix-borne, greyish-green or violet with the matrix consisting of sand and sludge and the balls mainly of granite, gneiss, dolomite and pebbles. The climate is just on the border of Northern Boreal and Arctic making this a location particularly sensitive to climate-induced vegetation change. The present vegetation is birch-light birch woodland as recorded by mapping for this project (Fig. 1).

Two sediment cores were taken from Nordvivatnet using either a modified Nesje piston or Multisampler corer, as described in (33). Nesje core sections were split longitudinally, with one half used for sampling. The intact core halves were photographed at high resolution and non-invasively scanned using X-ray fluorescence (XRF) and magnetic susceptibility (MS) at the Department of Geosciences, The Arctic University of Norway in Tromsø. XRF point measurements were taken every 5 mm at 10 kV using an Avaatech XRF core scanner, whereas we used a GEOTEK Multi Sensor Core Logger (MSCL-S) with a point sensor for MS logging. All XRF and MS measurement data are in SI Appendix Dataset S5. The remaining Nesje core halves and Multisampler core were used to take samples for loss-on-ignition (LOI), radiocarbon, sedaDNA, and pollen/spore analyses. All LOI and radiocarbon data are given in (3). A total of 18 radiocarbon dates from the Nesje core were used to inform the construction of an age-depth model, which was presented in (3) and SI Appendix Fig. S4. The Multisampler and Nesje cores were aligned using LOI profiles and two radiocarbon dates from the Multisampler core (3). Those excluded by the model were all at the base of the core where it was suspected slumping occurred into the lake in the Younger Dryas.

**Plant sedaDNA analysis.** The plant sedaDNA data set was generated either by (33) or here following the methods of that study. Briefly, we extracted DNA from 0.25-0.35 g of sediment and used DNA metabarcoding, targeting the vascular plant trnL p6-loop locus (54, 55), to generate our data set from 46 samples, from throughout the Nordvivatnet core (EG10), as well as 4 negative extraction controls. Of these samples, 29 of Holocene age were reported by (33), whereas the 17 Younger Dryas samples, of which one was extracted twice, are reported by (56; SI Appendix Dataset S9). We followed the bioinformatics pipeline of (3), which uses the ObiTools software package (57) and custom R scripts (available at https://github.com/Y-Lammers/MergeAndFilter) to filter the data and remove artifacts. We provide a tag-to-PCR replicate lookup identifier in SI Appendix Dataset S1. We identified barcode sequences that had 100% identity agreement with either of the ArctBorBryo (58-60) or EMBL (rl133), and/or PhyloNorway (61) nucleotide databases. We further removed identified sequences that 100% matched against two lists of potential false positives consisting of either known contaminants and exotics or synthetic sequences (3). We also filtered poorly performing samples based on metabarcoding success using technical and analytical quality (MTQ/MAQ) scores following (33) (SI Appendix Dataset S9). The final taxonomic assignment of the retained sequences was determined using regional botanical taxonomic expertise by Alsos. Note that these determinations are revised from (33) and are presented in SI Appendix Dataset S8. We followed the taxonomy of the Panarctic Flora and Lids Norsk Flora (62). If multiple sequences were assigned to the same taxon, then the data were merged using the sum of all reads and the maximum number of PCR replicates.

**Animal sedaDNA analysis.** We generated the animal sedaDNA data set from the same DNA extracts used above. We used the MamP007 primers (63) to amplify a 60–84-bp fragment of the mitochondrial 16S locus (excluding primer length), following the protocol described in (64) that includes the use of a dual-blocking strategy (tag-to-PCR replicate lookup in SI Appendix Dataset S1). PCR product cleaning, pooling, library preparation, and sequencing followed the protocols described above with library details in SI Appendix Dataset S1. Bioinformatic procedures prior to identification followed the same pipeline as the plant sedaDNA data set, except that we retained sequences that occurred in at least one PCR replicate in the entire data set. We identified barcode sequences that had ≥95% identity agreement to a reference database generated with the MamP007 primers applied to the EMBL (rl143) database using the ecoPCR tool (65), which we supplemented with a new 16S barcode sequence for Norwegian lemming (see SI Appendix text). Across the data set, this resulted in 166 retained barcode sequences (SI Appendix Dataset S3). We next collapsed barcodes with the same taxonomic assignment by summing read counts on a per PCR replicate basis and retaining the barcode(s) with the highest identity agreement. However, we did not collapse barcode sequences for human (*Homo sapiens*) and *Mysticeti*, due to high barcode sequence divergence within these taxa. We next revised the taxonomic assignments of the remaining 86 barcode sequences using taxonomic expertise (of Heintzman, Altenburger, Ehrich), reference database representation of related taxa, and biogeographic records from GBIF (<https://www.gbif.org>), with full justifications given in SI Appendix Dataset S2. 18 sequences assigned as human were removed as they matched to human nuclear mitochondrial DNA (NUMTs) and/or other non-mitochondrial loci (Not_16S). We then collapsed barcode sequences with the same taxonomic assignment, as above, resulting in 48 retained taxa. We assigned broad habitat data (marine, marine/freshwater, freshwater, terrestrial) to these taxa using the FishBASE and WoRMs databases (66). Two taxa had uncertain habitats (grey in Fig. 4; SI Appendix Fig. S8), whereas six taxa were assumed to be contaminants. These six were robustly identified and not expected in the region, have previously been reported as contaminants (67), and/or were present in the negative controls (SI Appendix Fig. S8). For each PCR replicate, we converted read counts to presence/absence data and calculated the proportion of positive detections from successful PCR replicates. PCR replicate success was defined as those with ≥10 reads in the raw read data (see SI Appendix Dataset S4).

**Pollen and Non-pollen Palynomorphs (NPPs).** A total of 36 subsamples of 1cm^3^ were prepared using a standard method of pollen and spore extraction (hydrofluoric treatment, acetolysis) and were mounted in glycerine at the Palaeoenvironmental Laboratory, University of Southampton. One *Lycopodium* tablet (n ≈ 12,489 grains) was added per sample to calibrate pollen concentration estimates. Where possible, at least 350 pollen grains of terrestrial taxa were identified per sample using standard taxonomic keys (68) and an extensive reference collection at the University of Southampton (SI Appendix Dataset S10). The pollen and spore data (73 samples at 7 cm intervals) presented from the Mortensnes on-site (Myr Ved Mortensnes) record is from earlier work by Høeg and reported in Norwegian in (40) and in SI Appendix Dataset S11.

For the identification and taxonomic assignment of NPPs we used a range of sources which included the NPP-ID Database (http://non-pollen-palynomorphs.uni-goettingen.de; 69), and the works by (70-72). An bespoke catalogue of types held at the Hugo deVries (HdV) laboratory with photographs from the multiple papers by van Geel was also used. All fungal and algal NPPs are assigned HdV codes as per the NPP-ID Database or other laboratory codes following (70) with the raw data in SI Appendix Dataset S12.

**Statistical analyses.** All statistical analyses were performed in R v.4.1.1 (73). The square-root transformed pollen percent data was used to establish pollen assemblage zones. We used presence/absence data of terrestrial plants while establishing stratigraphic zones for DNA-based vegetation reconstructions using the rioja package v.0.9.26 (74). We used Bray-Curtis distance for pollen and Jaccard distance for plant sedaDNA data and a constrained incremental sum of square (CONISS) approach while performing hierarchical clustering of samples. The broken stick model was used while selecting the appropriate number of vegetation zones. We also included an additional vegetation zone (5b, 2,200 BP) after visual inspection of the clusters but which got lower support according to the broken stick model. We treated the total number of taxa detected in a sample as taxonomic richness for both DNA and pollen data following (3). We evaluated temporal trends in taxonomic richness using a generalized additive model (GAM) with poisson distribution and log link. The changes in vegetation were evaluated by detrended correspondence analysis [(DCA)](https://paperpile.com/c/ECYfXn/TNas9/?prefix=DCA%2C) on weighted repeat data [(see details in 2)](https://paperpile.com/c/ECYfXn/aMOHK/?prefix=see%20details%20in). We considered DCA scores of the first axis as the measure of compositional change [(e.g. 3, 4)](https://paperpile.com/c/ECYfXn/Fs1y3+lpqQG/?prefix=e.g.,) and regressed against age of samples and oxygen isotope (δ¹⁸O) values from the North Greenland Ice Core Project (NGRIP)[(5)](https://paperpile.com/c/ECYfXn/ua3d4) as a climate proxy to assess how vegetation changed through time and during changes in past climatic conditions. We also analyzed how human population affected both the plant richness and composition by considering summed probability distribution (SPD), a measure of relative human population density, as the predictor in the GAM model (68). We used gaussian distribution with identity link while considering compositional change as the response variable in GAM. We accounted for residual temporal autocorrelation between consecutive samples by including a continuous-time first-order autoregressive process (CAR1) while fitting GAMs (GAM-CAR1 hereafter) using the “gamm” function following (75) when the predictor was sample age. The fitted lines for all models are based on the predicted values for 300 points covering the entire range of predictors. We generated a pointwise 95% confidence interval using critical value from the t distribution [(75)](https://paperpile.com/c/ECYfXn/1Rb1i). The GAM and GAM-CAR1 models were nearly identical (SI Appendix Fig. S9) and we included GAM models in the main text as they are simpler compared to GAM-CAR1 models.

**Norwegian lemming barcode sequence generation**

A new 16S barcode sequence was generated for Norwegian lemming (*Lemmus lemmus*). For this, a tail tissue specimen was used, which was derived from an individual collected from the Varanger Peninsula, northern Norway. DNA was extracted using a Qiagen DNeasy Blood & Tissue kit (Qiagen Norge, Oslo, Norway) following the manufacturer’s instructions. The extract was PCR amplified and sequenced using the same animal metabarcoding experimental setup described in the Methods. We used the same 16S bioinformatic pipeline to process the sequence data, up to and including the read identification step, with the exception that an unaltered version of the EMBL rl143 database was used. The read-dominant sequence is given below and was identified as Cricetidae with a 78% match, consistent with Norwegian lemming.

>Lemmus_lemmus_16S

TTAATTTCCTGGCCTAACTTATAAACTTATACCTCTACTGAACTAAATAGTAAAGTCATAGGCTAGCAATTTC

**Silhouette credits**

All silhouettes were downloaded from PhyloPic (<http://www.phylopic.org>). The frog image is by Michael Scroggie, from original photograph by John Bettaso, USFWS (original photograph in public domain); jellyfish by Mali'o Kodis, photograph by Ching (<http://www.flickr.com/photos/36302473@N03/>); polychaete worm by MC Hannon; cod by Milton Tan; sludge worm by B. Duygu Özpolat; goose by Rebecca Groom; earthworm by Luis Cunha; cow by Andreas Preuss/marauder; reindeer by mystica; human by NASA; and Bowhead whale, beaver, vole, and grouse are uncredited. Silhouettes are distributed under a Public Domain Mark 1.0 license (frog, polychaete worm, cod, Bowhead whale, cow, reindeer, human, beaver, vole, grouse), a Creative Commons Attribution 3.0 Unported license (<http://creativecommons.org/licenses/by/3.0/>; jellyfish, goose), or a Creative Commons Attribution-NonCommercial-ShareAlike 3.0 Unported license (<http://creativecommons.org/licenses/by-nc-sa/3.0/>; ​​earthworm, sludge worm). No changes were made to any of the silhouettes.

**SI Tables S1-S4**

Table S1. Summary of archaeological data for the Mortensness archaeological complex. All dates are on charcoal except * on shell with reservoir age not included, and ** on bone (see also Table S1). Dates in italics probably house re-use in later period. Sc is (44). House types (within 'features’) are now not commonly used but have been included here to allow comparison with the older literature.

| **Period** | **Sub-period, altitude** | **Features** | **Calibrated dates (2σ BP, median, Feature No., Lab Code)** | **Ecol./diet** | **Activities** | **Seasonality** | **Pop size** |
| --- | --- | --- | --- | --- | --- | --- | --- |
| **Old Stone Age (10000-5600 BP)** | Phase I 10000-9000 BP ~60-70 m asl | ~24 tent rings & floors | Not directly dated | - | Sea fishing.. | Spring-summer only? | Few small family units |
|  | Phase II  9000-7000 | First semi-subterranean pit-houses | 9133-9883 **9487** (R10 T2934)  7573-7670 **7614** (F8 Tra414)* | - | Sea fishing.. | Spring-summer only? | Single family dwellings, eastern+western peoples |
| Late Old Stone Age and early Younger Stone Age | Phase III 7000-5000 BP  26-19 m asl | 50+ small shell middens with small round pit houses (Karlebotn type) | 6192-7155 **6587** (R12 T6416)  6301-6673 **6495** (R12 T7219)  5144-5893 **5518** (R12 T7220)  *3724-4833* ***4273*** (R12 T6418) | Cod, harp seal… | Boat-fishing, sea cliffs gathering | Spring-early summer | Single family dwellings |
| **Late Younger Stone Age and Early Metal Age (5000-2000 BP)** | Period I 4400-3700 BP | Large Gressbakken type houses | - | - | - | - | semi-sedentary? |
|  | Period II-III 3700-2000 BP  18-12 m asl | 133+ pit dwellings incl. Mortensnes-type houses (100+) | 3775-4415 **4091** (R3, T7741)  3371-3876 **3605** (R3 T7740)  2883-3548 **3245** (R3 T7742)**  2155-2754 **2498** (R3, T675) | Whale, seal, porpoise | Boat-fishing, reindeer hunting | Year round? | Multi-family settlement(s) |
| **Iron Age and Medieval (2000-350 BP)** | Samí Iron Age | 47 gamme-type houses, meeting houses, graves (250-300), ritual sites | 1928-2463 **2201** (Grav2 T2934)  1730-2673 **2130** (R4 T674)  797-1176 **995** (R17, Sc p 93) | Sheep/goat, reindeer, cattle, pigs, ptarmigan, kittiwake, guillemot | Boat-fishing, reindeer herding?, pastoral and arable farming | Year round. | Multi-family settlement(s) and occasional large gatherings |
| **Historical (350-0 BP)** | Trade Post est. late 1700s | Sami ceremonial use and trade post (Andreas Georg Norđvi) | Lithograph 1871 CE (Friis) | - | Reindeer herding, fishing, sheep farming | Year round | A few families and occasional large gatherings and Norwegian traders |

Table S2. List of archaeological radiocarbon dates from the Mortensnes site. Dates were calibrated using OxCal and the IntCal13 calibration curve.

| **Site Code** | **Lab Code** | **Uncal. years BP** | **Years cal. BP median** | **Years cal. BP Range 2 sigma** | **Material** | **Source** |
| --- | --- | --- | --- | --- | --- | --- |
| R10 | T6415 | 8500±120 | 9487 | 9305-9883 | charcoal | Jørgensen comp. |
| F8 | Tra414 | 6758±32 | 7614 | 7573-7670 | Shell, no reservoir est. incl. | Jørgensen comp. |
| R12 | T6416 | 5770±190 | 6587 | 6321-7155 | charcoal | Jørgensen comp. |
| R12 | T7219 | 5700±90 | 6495 | 6399-6673 | charcoal | Jørgensen comp. |
| R12 | T7220 | 4800±120 | 5518 | 5326-5893 | charcoal | Jørgensen comp. |
| R12 | T6418 | 3860±200 | 4273 | 3934-4833 | charcoal | Jørgensen comp. |
| R3 | T7741 | 3730±110 | 4091 | 3910-4415 | charcoal | Jørgensen comp. |
| R3 | T7740 | 3360±110 | 3065 | 3456-3876 | charcoal | Jørgensen comp. |
| R3 | T7742 | 3060±120 | 3242 | 3017-3548 | bone | Jørgensen comp. |
| R3 | T675 | 2420±120 | 2498 | 2348-2754 | charcoal | Jørgensen comp. |
| Grav 2 | T2934 | 2210±100 | 2201 | 2104-2463 | charcoal | Jørgensen comp. |
| R4 | T674 | 2150±150 | 2130 | 1946-2673 | charcoal | Jørgensen comp. |
| R17 | ? | 1080±70 | 995 | 924-1176 | - | Schanche 1988 (p.93) |
| Side D | ? | 600±70 | 598 | 545-669 | - | Schanche 1988 |

Table S3. The synanthropic species used for the interpretation of the anthropogenic influence, with comments on their relationships to habitats and human activity.

| **Taxon** | **English common names** | **Pollination** | **Date of first appearance** | **Subsequent record** | **Comments** |
| --- | --- | --- | --- | --- | --- |
| *Arabis* (alpine?) | rockcress | insects | 4100 | Sporadic (2 adjacent samples) | alpine, damp screes & gravel, calcareous |
| *Ribes* | red/blackcurrent, ripps | Insects, bees | 2900 | Then almost continuous | Cultivated since 11^th^ C AD but also native to N Europe |
| *Pedicularis lapponica* | Lapland louswort | insects | 4300 | Presence then sporadic | Mountain birch woodlands, streams, meadows but grazing sensitive (27)  also common in tundra heath |
| *Vicia* cf. *cracca* | Cow vetch | Insects, bees, flies | 5500 | Peak then continued presence | synanthrope, associated with grasslands, grazed areas, hay meadows, and habitation  nitrogen fixating legume |
| *Anthriscus sylvestris* | Cow parsley, wild chervil, keck | Insects (flies in Svalbard) | 1700 | Peak until 3200 then sporadic | Strong synanthrope, associated with grazed areas and habitation |
| *Bartsia* (*alpina*?) | Alpine bartsia, velvetbells | Insects particularly bees | 3600 | Then continuous | Associated with pastures but sensitive to high grazing pressure |
| *Epilobium* | willowherb, fireweed | insects | 3600 | Single level then reappearance at 1600 | Strong synanthrope, associated with grazed areas and habitation |
| *Saussurea alpina* | Alpine saw-wort | insects | 6500 | Steady increase | Screes and open ground, stream banks, meadows  tolerant of disturbance |
| *Trollius* *europaeus* | globe flower | insects | 7000 | Continued presence | Damp habitats, stream banks, lake margins, meadows and open woodlands |

Table S4. Summary statistics for generalized additive models (GAMs). Richness calculation was based on both the pollen and DNA data. The first axis score of detrended correspondence analysis (DCA) based on DNA data was extracted to represent plant composition. edf: effective degrees of freedom; Dev.exp: deviance explained; Phi (ɸ): autocorrelation coefficient; adj.R.sq.: adjusted R square.

| Response | Predictor | edf | Test | Test score | p-value | Dev.exp (%) | adj.R.sq |
| --- | --- | --- | --- | --- | --- | --- | --- |
| DCA1 | NGRIP δ¹⁸O | 4.88 | F | 85.30 | < 0.0001 | 94.40 | 0.94 |
|  | Sample age | 7.00 | F | 116.40 | < 0.0001 | 97.30 | 0.97 |
|  | SPD | 3.82 | F | 11.00 | < 0.0001 | 72.30 | 0.67 |
| DNA richness | Sample age | 3.66 | ꭕ2 | 111.90 | < 0.0001 | 77.00 | 0.79 |
|  | SPD | 2.34 | ꭕ2 | 24.66 | < 0.0001 | 57.90 | 0.55 |
| Pollen richness | Sample age | 2.79 | ꭕ2 | 11.08 | < 0.05 | 35.30 | 0.30 |

**References not in main paper.**

54. Taberlet, P, Coissac, E, Pompanon, F, Gielly, L, Miquel, C et al. (2007) Power and limitations of the chloroplast trnL (UAA) intron for plant DNA barcoding. *Nucleic Acids Res.* 35: e14.

55. Taberlet, P, Bonin, A, Zinger, L, Coissac, E (2018) *Environmental DNA: For Biodiversity Research and Monitoring* (Oxford Scholarship Online, Oxford)

56. Alsos I.G., Rijal, D.P., Ehrich, D., Karger, D.N., Yoccoz, N.G., Heintzman, P.D., Brown, A.G., Lammers, Y., Pellissier, L., Alm, T., Bråthen, K.A., Coissac, E., Føreid Merkel, M.K., Alberti, A., Denoeud, F., Bakke, J.,PhyloNorway Consortium. 2022. Postglacial species arrival and diversity buildup of northern ecosystems took millennia. *Sci. Adv.* 8, eabo7434

57. F. Boyer, F, Mercier, C, Bonin, A, Le Bras, Y, P. Taberlet, E et al. (2016) OBITOOLS: A unix-inspired software package for DNA metabarcoding. *Mol. Ecol. Resour.* 16: 176–182

58. Sønstebo, JH, Gielly L, Brysting A, Reidar E, Edwards M et al. (2010) Using next-generation sequencing for molecular reconstruction of past Arctic vegetation and climate. *Mol Ecol Res* 10: 1009-1018.

59. Willerslev, E, Davison, J, Moora, M, Zobel, M, Coissac, M et al. (2014) Fifty thousand years of Arctic vegetation and megafaunal diet. *Nature* 506, 47–51.

60. Soininen, M, Gauthier, G, Bilodeau, F, Berteaux, D, Gielly, L (2015) Highly overlapping winter diet in two sympatric lemming species revealed by DNA metabarcoding. *PLOS ONE* 10: e0115335.

61. Alsos, IG, Sjögren, P, Brown, AG, Gielly, L, Merkel, MKF (2020) Last Glacial Maximum environmental conditions at Andøya, northern Norway; evidence for a northern ice-edge ecological “hotspot”. *Quat. Sci. Rev.* 239:106364.

62. Elven, R, Alm, T,Berg, T, Båtvik, JII, Fremstad, E et al. (2005) *Norsk flora* (Det Norske Samlaget, Oslo).

63. Giguet-Covex, C, Pansu, J, Arnaud, F, Rey, P-J, Griggo, C, et al. (2014) Long livestock farming history and human landscape shaping revealed by lake sediment DNA. *Nat Coms* 5:3211.

64. Garcés-Pastor, S, Lavergne, S, Coissac, E, Lammers, Y, Wangensteen, OW, Theurillat, J-P, Schwörer, C, Brown, AG, Tinner, W, Rey, F, Heiri, O, Heer, M, Rutzer, A, Walsh, K, Heintzman, PD, Goslar, T, Alsos, IG (In Press). Good plant sedaDNA indicators allow robust vegetation response to temperature shifts along the Holocene in Central Alps. *Nat. Coms.*

65. Ficetola, G, Coissac, E, Zundel, S, Riaz, T, Shehzad, W et al. (2010) An In silico approach for the evaluation of DNA barcodes. *BMC Genomics* 11, 434.

66. Horton, T, Kroh, A. et al. (2021). World Register of Marine Species. Available from https://www.marinespecies.org at VLIZ. Accessed 2021-09-10. doi:10.14284/170

67. Leonard, JA, Shanks, O, Hofreiter, M, Kreuz, E, Hodges, L et al. (2007). Animal DNA in PCR reagents plagues ancient DNA research. *J Arch Sci* 34: 1361-1366.

68. Fægri and Iversen, 1989. *Textbook of Pollen Analysis.* (J Wiley, Chichester).

69. Shumilovskikh L.S., Shumilovskikh E.S., Schlütz F., van Geel B. (2021) NPP-ID - Non-Pollen Palynomorphs Image Database as a research and educational platform. Vegetation History and Archaeobotany, in press; DOI: 10.1007/s00334-021-00849-8.

70. Miola A (2012) Tools for Non-Pollen Palynomorphs (NPPs) analysis: A list of Quaternary NPP types and reference literature in English language (1972-2011). Review of Palaeobotany and Palynology 186:142-161.

71. Van Asperen EN, Perrotti A, Baker A (2021) Coprophilous fungal spores: non-pollen palynomorphs for the study of past megaherbivores. In: Marret F., O’Keefe J., Osterloff P., Pound M., Shumilovskikh L. (2021) Applications of Non-Pollen Palynomorphs: from Palaeoenvironmental Reconstructions to Biostratigraphy. Geological Society, London, Special Publications, 511,

72. Marret F., O’Keefe J., Osterloff P., Pound M., Shumilovskikh L. (2021) Applications of Non-Pollen Palynomorphs: from Palaeoenvironmental Reconstructions to Biostratigraphy. Geological Society, London, Special Publications, 511.

73. R Core Team (2021). R: A language and environment for statistical computing. R Foundation for Statistical Computing, Vienna, Austria. URL https://www.R-project.org/.

74. Simpson, GL (2018). Modelling Palaeoecological Time Series Using Generalised Additive Models. *Front Ecology Evol* 6:149.

75. Juggins, S. (2020) rioja: Analysis of Quaternary Science Data, R package version (0.9-26). (https://cran.r-project.org/package=rioja).
